# PARP1 regulates DNA damage-induced nucleolar-nucleoplasmic shuttling of WRN and XRCC1 in a toxicant and protein-specific manner

**DOI:** 10.1038/s41598-019-46358-7

**Published:** 2019-07-11

**Authors:** Sebastian Veith, Andrea Schink, Marina Engbrecht, Matthias Mack, Lisa Rank, Pascal Rossatti, Mariam Hakobyan, Denise Goly, Tanja Hefele, Marco Frensch, Arthur Fischbach, Alexander Bürkle, Aswin Mangerich

**Affiliations:** Molecular Toxicology Group, Department of Biology, University of Konstanz, Konstanz, Germany

**Keywords:** Cellular imaging, Nucleolus, PolyADP-ribosylation

## Abstract

The prime function of nucleoli is ribogenesis, however, several other, non-canonical functions have recently been identified, including a role in genotoxic stress response. Upon DNA damage, numerous proteins shuttle dynamically between the nucleolus and the nucleoplasm, yet the underlying molecular mechanisms are incompletely understood. Here, we demonstrate that PARP1 and PARylation contribute to genotoxic stress-induced nucleolar-nucleoplasmic shuttling of key genome maintenance factors in HeLa cells. Our work revealed that the RECQ helicase, WRN, translocates from nucleoli to the nucleoplasm upon treatment with the oxidizing agent H_2_O_2_, the alkylating agent 2-chloroethyl ethyl sulfide (CEES), and the topoisomerase inhibitor camptothecin (CPT). We show that after treatment with H_2_O_2_ and CEES, but not CPT, WRN translocation was dependent on PARP1 protein, yet independent of its enzymatic activity. In contrast, nucleolar-nucleoplasmic translocation of the base excision repair protein, XRCC1, was dependent on both PARP1 protein and its enzymatic activity. Furthermore, gossypol, which inhibits PARP1 activity by disruption of PARP1-protein interactions, abolishes nucleolar-nucleoplasmic shuttling of WRN, XRCC1 and PARP1, indicating the involvement of further upstream factors. In conclusion, this study highlights a prominent role of PARP1 in the DNA damage-induced nucleolar-nucleoplasmic shuttling of genome maintenance factors in HeLa cells in a toxicant and protein-specific manner.

## Introduction

The nucleolus is a dynamic, non-membrane bound compartment of the nucleus^1,2^. Its prime function is ribosome biogenesis, comprising rRNA synthesis and ribosome assembly. In addition, the nucleolus has recently emerged as a key regulator of other cellular processes including DNA damage response^3^. For example, upon DNA damage, many proteins, including several genome maintenance factors, shuttle between the nucleolus and the nucleoplasm. However, the underlying molecular mechanisms are still poorly understood. In this study, we investigated how PARP1 and PARylation contribute to nucleolar-nucleoplasmic shuttling of two exemplary-chosen genome maintenance factors, i.e. WRN and XRCC1, upon several different forms of genotoxic stress.

The nucleolus has a tripartite organization, consisting of the fibrillar center (FC), the dense fibrillar component (DFC), and the granular component (GC), which is surrounded by peri-nucleolar heterochromatin. These structures are formed around genomic nucleolar organizer regions (NOR), harboring clusters of 200–400 rRNA genes localized as tandem repeats at the short arms of the five acrocentric chromosomes, which are actively transcribed by RNA polymerase I. After transcription and processing of rRNA, ribosomal proteins cooperatively assemble to form the pre-40S and pre-60S ribosomal subunits that are exported to the cytoplasm to produce mature 80S ribosomes^1,2^. Many signaling pathways associated with growth factors and nutrient availability regulate rRNA synthesis, and therefore the general metabolic activity of a cell and nucleolar function are tightly and reciprocally connected. In the last years, several proteomic studies demonstrated that the nucleolus harbors hundreds of non-ribosomal proteins^4,5^, which are associated with a number of non-canonical nucleolar functions, including cell cycle regulation, cell proliferation, and stress response^6^. In agreement with this, nucleolar functions have been implicated in complex physiological and pathophysiological conditions on the organismic level, such as cancer and aging^7–10^. Furthermore, the nucleolus and nucleolar mechanisms have been suggested as targets for cancer therapy^11,12^.

The role of the nucleolus in genotoxic stress response and genome maintenance is an emerging research topic, which has been comprehensively reviewed recently^13–16^. In total >150 DNA repair proteins localize to nucleoli under non-stress conditions^16^ and at least some of them undergo DNA damage-specific nucleolar-nucleoplasmic shuttling upon induction of genotoxic stress^17^. It is a matter of active investigations if the nucleolus acts as a mere reservoir and storage site for genome maintenance factors that operate in the nucleoplasm upon their nucleolar release, or if such factors indeed play specific roles in nucleolar processes, e.g. in the repair of damaged rDNA sequences or in DNA repair-unrelated functions such as RNA metabolism - or both. Furthermore, at present it is not fully understood by which mechanisms nucleolar proteins translocate from the nucleolus to the nucleoplasm and vice versa^3,18^.

Poly(ADP-ribosyl)ation (PARylation) is an essential post-translation modification in most eukaryotic organisms. It is mediated by members of the family of poly(ADP-ribose) polymerases (PARPs) by using NAD^+^ as a substrate to generate the nucleic acid-like, highly negatively charged biopolymer poly(ADP-ribose) (PAR), which can be composed of molecules of heterogenous chain lengths and branching frequencies. The major DNA damage-induced PARP is PARP1 (also known as ARTD1), which is mainly activated by DNA single and double strand breaks and which accounts for >90% of DNA damage-induced PARylation^19^. Apart from covalent PARylation, PAR can bind non-covalently to target proteins via several high-affinity PAR-binding modules^20,21^. It is important to note that cellular PARylation is highly dynamic and fully reversible, since several PAR-catabolizing enzymes exist^22^. By this, PARP1 and PARylation regulate enzymatic activities as well as association and dissociation dynamics of protein complexes in a spatio-temporal manner. On a cellular level, PARP1 participates in several DNA repair and DNA damage response mechanisms, yet it also exhibits functions beyond those, such as chromatin remodeling, transcription, and regulation of cell cycle and cell death^23^. On the organismic level, PARP1 has been implicated in various physiological and pathophysiological conditions, such as carcinogenesis, aging, and inflammatory diseases. Importantly, PARP inhibitors have attracted much attention in the last years for their use in cancer chemotherapy, either by their use as chemosensitizers in combination with classical DNA-damaging chemotherapeutics or as stand-alone drugs in homologous-deficient tumors following the concept of synthetic lethality^24,25^.

It has been estimated that about 40% of PARP1 molecules of a cell localize to nucleoli and upon DNA damage PARP1 can translocate to the nucleoplasm^26–28^. Interestingly, in *Drosophila*, PARP is essential for the development of functional nucleoli by stabilizing the general nucleolar architecture through the formation of complexes with nucleolar proteins, such as fibrillarin and nucleophosmin^28,29^. Functionally, *Drosophila* PARP participates in ribosomal biogenesis by regulating precursor rRNA processing, post-transcriptional modification, and pre-ribosome assembly^30^. Furthermore, it was demonstrated that PARP1-mediated PARylation of TIP5, which is part of the nucleolar remodeling complex (NoRC), promotes the silencing of rDNA chromatin during replication, thereby uncovering a mechanism by which PARP1 ensures that silent rDNA regions are properly inherited^31,32^.

Here, we addressed the question if and how PARP1 and PARylation regulate the subnuclear localization dynamics of two key factors of genome maintenance, i.e. the RECQ helicase, WRN, and the base excision repair (BER) protein, XRCC1, upon induction of genotoxic stress. It was previously reported that both proteins undergo nucleolar-nucleoplasmic shuttling upon various forms of DNA damage, such as treatment with hydrogen peroxide (H_2_O_2_) and camptothecin (CPT)^18,33–40^. Previous studies also suggested that post-translational modifications, such as acetylation and phosphorylation^34,36^, are involved in these localization dynamics. Despite these intriguing initial mechanistic insights, we still lack a detailed understanding of the underlying molecular mechanisms of nucleolar-nucleoplasmic trafficking of these proteins. Since WRN and XRCC1 physically and functionally interact with PARP1/PAR, and both factors are tightly regulated by the PARP1/PAR system^33,41–45^, we reasoned that their subnuclear localization dynamics might be regulated by PARP1 and/or PARylation.

## Material and Methods

### Cell culture, transfection and genotoxic treatment

HeLa Kyoto WT and *PARP1* knock-out (KO) cells were cultivated under standard conditions (37 °C, 95% humidity, and 5% CO_2_) as described previously^19^. Treatments with genotoxic agents, i.e., hydrogen peroxide (H_2_O_2_, Merck-Millipore), 2-chloroethyl ethyl sulfide (CEES, Sigma Aldrich), and camptothecin (CPT, Sigma-Aldrich) were conducted as described in individual figures.

Olaparib and ABT888 were purchased from Selleckchem; (±)-gossypol from Sigma Aldrich. For transfection experiments, cells were transiently transfected with eGFP-tagged expression constructs for PARP1^19^ and WRN (pEGFP::C3WRN plasmid was a kind gift of V. Bohr, NIA/NIH, Baltimore) using Effectene reagent (Qiagen), essentially as described previously^19^. Briefly, 6 h before cells were transfected, 1 × 10^5^ *PARP1* KO cells per well were seeded in 12-well plates and then transfected with corresponding DNA constructs. Subsequent steps were conducted according to the manufacturer’s instructions. The day after transfection, medium was exchanged, and one to two days after transfection, immunofluorescence experiments were performed.

### Immunofluorescence staining

For immunofluorescence staining, 1 × 10^6^ WT and 1.2 × 10^6^ *PARP1* KO cells were seeded per well in 12-well plates containing 15-mm coverslips. Twenty-four hours after seeding, cells were treated with genotoxic agents as indicated or left untreated. After treatment, medium was aspirated, and cells were washed once with 1 ml PBS. Then coverslips were transferred to fresh 12-well plates and cells were fixed in 1 ml of 4% formaldehyde for 20 min at room temperature (RT). Formaldehyde solution was aspirated and fixation was stopped by adding 1 ml of 100 mM glycine in PBS. After washing for 5 min in PBS, cells were incubated with 0.4% Triton X-100 for 3 min, followed by another washing step for 5 min in PBS. Samples were incubated in PBSMT for 1 h at room temperature while shaking to saturate binding of unspecific epitopes. Subsequently, coverslips were transferred to a humid chamber and the respective primary antibody was added, i.e., rabbit-anti-XRCC1 (Enzo Life Sciences, 1:750–1:1000), rabbit-anti-WRN (H-300, Santa Cruz, 1:50; ab200, Abcam, 1:800), mouse-anti-WRN (1:2000, kind gift of Vilhelm Bohr, NIA/NIH), mouse-anti-fibrillarin (Abcam, 1:800–1:1000), mouse-anti-nucleolin (Santa Cruz, 1:500), mouse-anti-GFP (Roche, 1:150), mouse-anti-PAR (10H, 1:300)^46^, mouse-anti-PARP1 (CII-10, 1:300, kind gift of G. G. Poirier, Quebec, Canada), rabbit-anti-BLM (Santa Cruz, 1:100), mouse-anti-RECQL1 (Santa Cruz, 1:400), rabbit-anti-RECQL4 (Proteintech, 1:200), and rabbit-anti-RECQL5 (Sigma-Aldrich, 1:400). Cells were incubated with the primary antibody at 4 °C overnight or at 37 °C for 1 h. Afterwards, cells were again washed thrice for 10 min in PBS and then incubated with the respective secondary antibody for 1 h at 37 °C, i.e., goat-anti-rabbit-IgG-Alexa488 (ThermoFisher, 1:400), goat-anti-mouse-IgG-Alexa488 (ThermoFisher, 1:400), goat-anti-rabbit-IgG-Alexa546 (ThermoFisher, 1:400–1:600), goat-anti-mouse-IgG-Alexa647 (ThermoFisher, 1:400). Subsequently, cells were washed thrice for 10 min in PBS and samples were incubated with 0.1 mg/µl Hoechst 33342 for 5 min at RT for staining of nuclei. Thereafter, samples were washed again thrice for 5 min each and coverslips were mounted on glass slides using Aqua Polymount (Polysciences). Samples were dried for at least 24 h and analyzed by confocal laser scanning microscopy.

### Confocal microscopy

Microscopy was performed on a Zeiss LSM700 confocal microscope at the Bioimaging Center of the University of Konstanz. Specimen were analyzed with a Plan-Apochromat 63×/1.4 Oil Ph3 M27 objective. Hoechst33342 (ThermoFisher) was excited with a 405-nm diode, Alexa Fluor 488 with a 488-nm diode, Alexa Fluor 546 with a 555-nm diode and Alexa Fluor 647 with a 637-diode. The pinhole of the channel with the longest wavelength was set to 1 airy unit (AU), the resulting pinhole diameter was adjusted manually to the same size in the other channels to achieve identical focal planes. Master gain, master offset and laser intensities were adjusted for independent experiments to obtain optimal results. For each condition, images of >90 cells were acquired at a resolution of 1152 × 1152 pixels.

### Image analysis

Manual categorized image analysis was performed in the initial phase of this project and later for experiments, when for technical reasons no fibrillarin staining as a nucleolar marker could be performed. Microscopic images were blinded prior to analyses. For each condition, >90 cells were analyzed. To this end, cells were categorized into one of three categories. Thus, cells with stronger fluorescence intensity within the nucleoli compared to the nucleoplasm were classified as nucleoli with ‘*strong*’ WRN, XRCC1, or PARP1 signal; cells with homogenous fluorescence distribution were classified as nucleoli with ‘*medium*’ signal; and cells with lower fluorescence intensity within the nucleoli than in the nucleoplasm as nucleoli ‘*without*’ protein signal.

When possible, automated image analysis using a newly developed KNIME workflow was used for the analysis of the subnuclear localizations of WRN and XRCC1. The image-processing workflow calculates the ratio of the mean fluorescence intensities in nucleolar areas (A_nucleoli_) and nuclear areas excluding nucleolar areas (A_nuclei-nucleoli_) for each condition [(mean fluorescence intensity A_nucleoli_)/(mean fluorescence intensity A_nuclei-nucleoli_)]. The nuclear area is determined by Hoechst 33342 staining, while the nucleolar area A_nucleoli_ is determined by fibrillarin staining. A ratio above ‘1’ indicates a higher mean fluorescence intensity within the nucleoli compared to the mean fluorescence intensity in nucleoplasm (A_nuclei-nucleoli_), which corresponds to the situation where proteins accumulate in nucleoli (‘*strong’* signal). A ratio below ‘1’ indicates higher mean fluorescence intensities in A_nuclei-nucleoli_. For better illustration in figures, the value of ‘1’ was subtracted from the ratios.

### Determination of H_2_O_2_ scavenging activity

To determine the H_2_O_2_ scavenging activity of gossypol, a spectrophotometric method described by Mukhopadhyay *et al*. was performed in a 96-well plate^47^. First, 75 µM ferrous ammonium sulphate (Sigma-Aldrich) was mixed with gossypol in concentrations of 47.2 nM to 94.4 µM or, as a positive control, with ascorbic acid in concentrations of 7.813 µg/ml and 1000 µg/ml. A reaction mix without any test compound and without H_2_O_2_ served as a negative control. After addition of H_2_O_2_ to a final concentration of 94.4 µM the mixture was incubated for 5 min in the dark. Thereafter, 45.3 µM 1,10-phenanthroline (Sigma-Aldrich) was added, followed by a 10-min incubation period in the dark to allow tri-phenanthroline complex formation. Absorbance was measured with a spectrophotometer at 510 nm. H_2_O_2_ scavenging activity was calculated by dividing the absorbance of the sample by the absorbance of the negative control, after subtraction of the background signal.

### Statistical analysis

Experiments were performed in replicate numbers as indicated, data evaluated with GraphPad Prism 6, and statistical tests performed as indicated. ‘*’ Indicates P ≤ 0.05, ‘**’ indicates P ≤ 0.01, ‘***’ indicates P ≤ 0.001.

## Results

**Figure 1:**
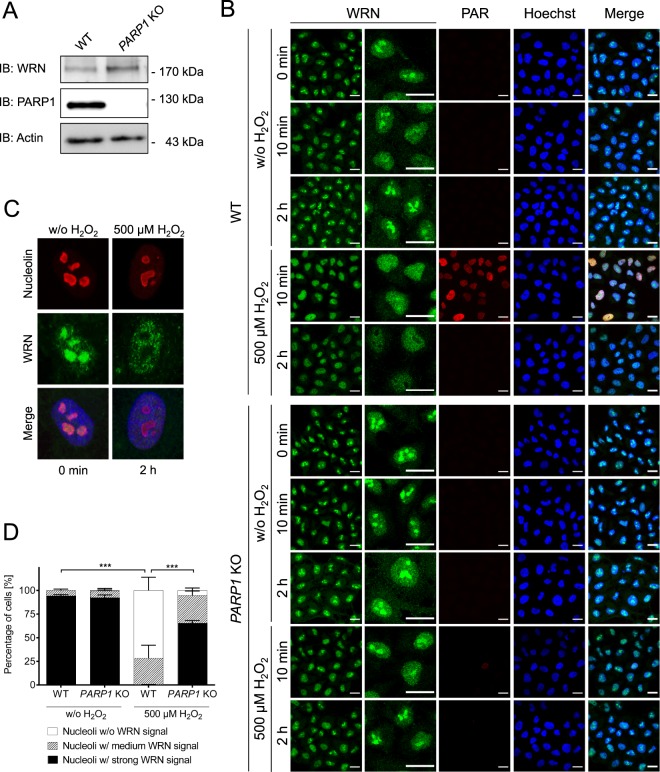
H_2_O_2_-induced WRN translocation from nucleoli to the nucleoplasm is PARP1-dependent. (**A**) Western blot verifying the *PARP1* KO on the protein level of HeLa cells used in this study and as described previously^19^. Full-length blots are shown in Suppl. Fig. 13. (**B**) HeLa WT and *PARP1* KO cells were left untreated or treated with 500 µM H_2_O_2_ for periods as indicated. Samples were then subjected to immunofluorescence staining for WRN and PAR and analyzed by confocal microscopy. Representative images of three independent experiments are shown. The second column displays magnified sections of images of the first column. Brightness and contrast were adjusted for better visibility. Scale bars represent 20 µm. (**C**) Co-staining of WRN and the nucleolar marker protein nucleolin verifying the nucleolar localization of WRN under untreated conditions. (**D**) Categorized image analysis of WRN nucleolar localization as described in the ‘material and methods’ section of three independent experiments. Cells were treated with H_2_O_2_ for 2 h. Data represent means ± SEM. Statistical analysis was performed by a Chi-square test.

To test the hypothesis if PARP1 regulates the nucleolar-nucleoplasmic shuttling of WRN and XRCC1 upon induction of genotoxic stress, we used HeLa WT and genetically modified HeLa *PARP1* KO cells (Fig. 1A), which have recently been generated and comprehensively characterized^19^. By combining this genetic system with pharmacological PARP inhibition, our approach allows to dissect the enzymatic and non-enzymatic functions of PARP1 itself as well as PARylation in general. We performed a detailed analysis of the subnuclear localization dynamics of WRN and XRCC1 upon treatment with different genotoxic agents using confocal microscopy and fully automated quantitative image analyses. As genotoxic stimuli we used the oxidizing agent, H_2_O_2_, the alkylating agent, 2-chloroethyl ethyl sulfide (CEES), and the topoisomerase I inhibitor, CPT.

### WRN is released from nucleoli upon genotoxic treatment in a PARP1-dependent manner

**Figure 2:**
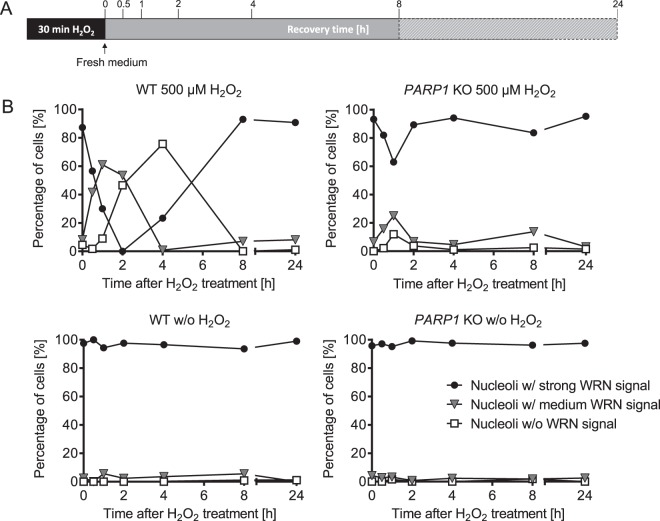
H_2_O_2_-induced PARP1-dependent WRN translocation from nucleoli to the nucleoplasm is fully reversible. (**A**) Treatment schedule of HeLa WT and *PARP1* KO cells. (**B**) HeLa WT and *PARP1* KO cells were treated as indicated and subjected to immunofluorescence staining for endogenous WRN and analyzed by confocal microscopy as described in Fig. 1. Shown are categorized data analyses of nucleolar WRN localization as described in the ‘material and methods’ section. Representative original microscopic imaging data are shown in Suppl. Fig. 2.

**Figure 3:**
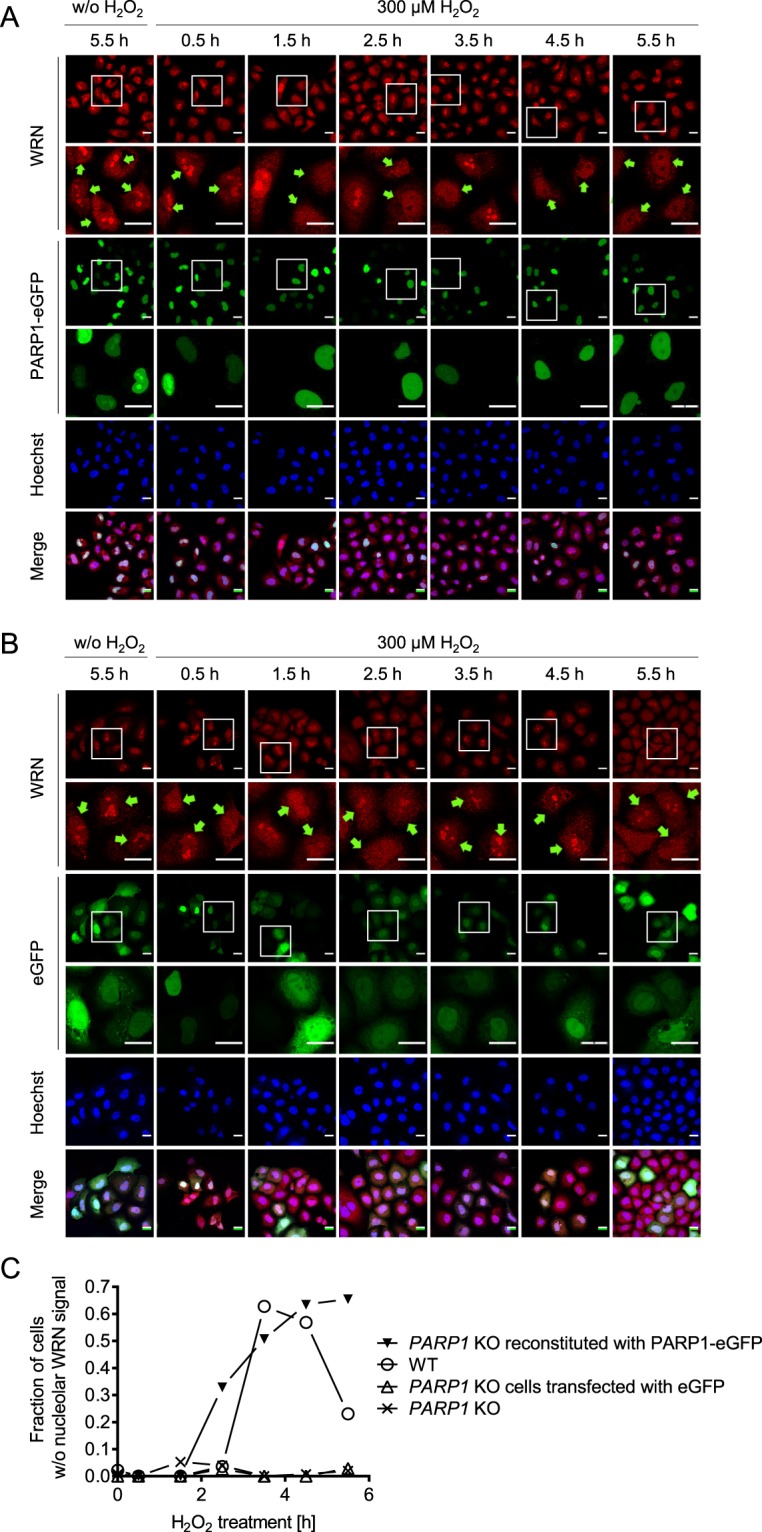
WRN localization in *PARP1* KO cells reconstituted with eGFP-tagged PARP1-WT and treated with H_2_O_2_. After transfection with an eGFP-tagged PARP1 construct (**A**) or an eGFP vector control (**B**), HeLa *PARP1* KO cells were treated with 300 µM H_2_O_2_ for periods as indicated. Samples were subjected to immunofluorescence staining for WRN and subsequently analyzed by confocal microscopy. White frames indicate magnified image sections. Transfected cells are marked by green arrows. (**C**) Categorized analysis of nuclear WRN localization of data as shown in A. Quantification was performed as described in the ‘material and methods’ section. For better illustration, only the relative fractions of cells without nucleolar WRN signal are depicted. Brightness and contrast were adjusted for better visibility. Scale bars represent 20 µm.

To study if WRN translocation from nucleoli to nucleoplasm is PARP1-dependent, we treated WT and *PARP1* KO cells with the strong PARylation inducer H_2_O_2_^19^. As expected, this led to a strong PARylation response 10 min after treatment in WT cells, but not in *PARP1* KO cells, confirming that the bulk of PARylation activity upon H_2_O_2_ treatment is mediated by PARP1 (Fig. 1B). Consistent with previous studies^34–40^, WRN localized to nucleoli of HeLa cells under unstressed conditions (Fig. 1C). To evaluate the nucleolar localization of WRN we blinded samples and categorized cells into three groups: (*i*) Cells with stronger fluorescence intensity within the nucleoli compared to the nucleoplasm were classified as nucleoli with ‘*strong*’ WRN signal; (*ii*) cells with homogenous fluorescence distribution were classified as nucleoli with ‘*medium*’ signal; and (*iii*) cells with lower fluorescence intensity within the nucleoli than in the nucleoplasm as nucleoli ‘*without*’ WRN signal (for detailed description refer to the ‘material and methods’ section). (*N.B*. We used this type of manual image evaluation in the initial phase of the project and whenever necessary for technical reasons, such as species incompatibilities of antibody combinations. For all other experiments we applied a fully automated image analysis as described below). Endogenous WRN, which accumulated in nucleoli under normal, non-stressed cell culture conditions, was completely released from nucleoli in WT cells 2 h after H_2_O_2_ treatment (Fig. 1B,D). In contrast, in *PARP1* KO cells subnuclear WRN localization was hardly affected by H_2_O_2_ treatment, demonstrating that PARP1 acts as a key factor in the H_2_O_2_-induced release of WRN from nucleoli. To test if other RECQ helicases, i.e. RECQ1, BLM, RECQ4, and RECQ5, are present in HeLa cell nucleoli we analyzed the subnuclear distribution of endogenously expressed RECQ proteins using commercially available antibodies. As it is evident from Suppl. Fig. 1, none of the other RECQ helicases could be found in nucleoli in our experimental system. Therefore, we focused on WRN for subsequent analyses. To examine if the H_2_O_2_-induced release of WRN from nucleoli is reversible or goes along with a complete and persistent exclusion from nucleoli, we performed long-term experiments. To this end, cells were treated for a defined period of 30 min with 500 µM H_2_O_2_, and afterwards were allowed to recover in fresh medium for up to 24 h. (*N.B*. please note that this experimental setup is different than the one applied in the short term treatment shown in Fig. 1, in which H_2_O_2_ was not removed after 30 min). Figure 2 and Suppl. Fig. 2 demonstrate that 4 h after H_2_O_2_ treatment, WRN started relocating to nucleoli in WT cells. By 8 h after H_2_O_2_ treatment, WRN had fully relocated to nucleoli. Consistent with data from Fig. 1, only a slight release of WRN was observed in *PARP1* KO cells upon H_2_O_2_ treatment, even at later time points. To verify that the observed effects are indeed WRN-specific and not related to any unknown technical artefact, such as potential antibody cross reactivities, we additionally analyzed WRN translocation using a WRN antibody raised against an alternative epitope (Suppl. Fig. 3). Furthermore, we expressed WRN ectopically in WT and *PARP1* KO cells using a GFP-tagged WRN construct and subsequently performed immunofluorescence staining against GFP (Suppl. Fig. 4). Both approaches validated the result of Figs. 1 and 2, showing that WRN release from nucleoli is strongly reduced in *PARP1* KO cells compared to WT cells. To exclude that the observed effects are related to any potential genetic off-target effects that might have occurred during the generation of *PARP1* KO cells, we reconstituted *PARP1* KO cells with a GFP-tagged PARP1-WT construct [as described previously^19^]. Fully consistent with the former experiments, WRN release from nucleoli could be completely rescued in cells reconstituted with PARP1 (Fig. 3).

Next, we were interested if PARP1-dependent WRN release from nucleoli also occurs after treatment with other genotoxic agents, i.e., the sulfur mustard analogue and alkylating agent, CEES, and the topoisomerase I inhibitor, CPT. Both agents induced the release of WRN from nucleoli (Suppl. Figs. 5 and 6). In case of CEES treatment, WRN release from nucleoli was partially PARP1-dependent, although the effect was not as pronounced as after H_2_O_2_ treatment (Suppl. Fig. 5). Interestingly, the CPT-induced release of WRN from nucleoli was independent of PARP1 (Suppl. Fig. 7). These results indicate toxicant-specific mechanisms that lead to a translocation of DNA repair factors, such as WRN, from nucleoli. Since the PARP1-dependent effect on WRN translocation was most prominent upon H_2_O_2_ treatment, we focused on this toxicant in the following experiments. In summary, these data demonstrate that WRN shuttles from nucleoli to the nucleoplasm upon induction of various forms of genotoxic stress and that this shuttling is dependent on PARP1 in a toxicant-specific manner.

### XRCC1 is released from nucleoli upon genotoxic treatment in a PARP1-dependent manner

**Figure 4:**
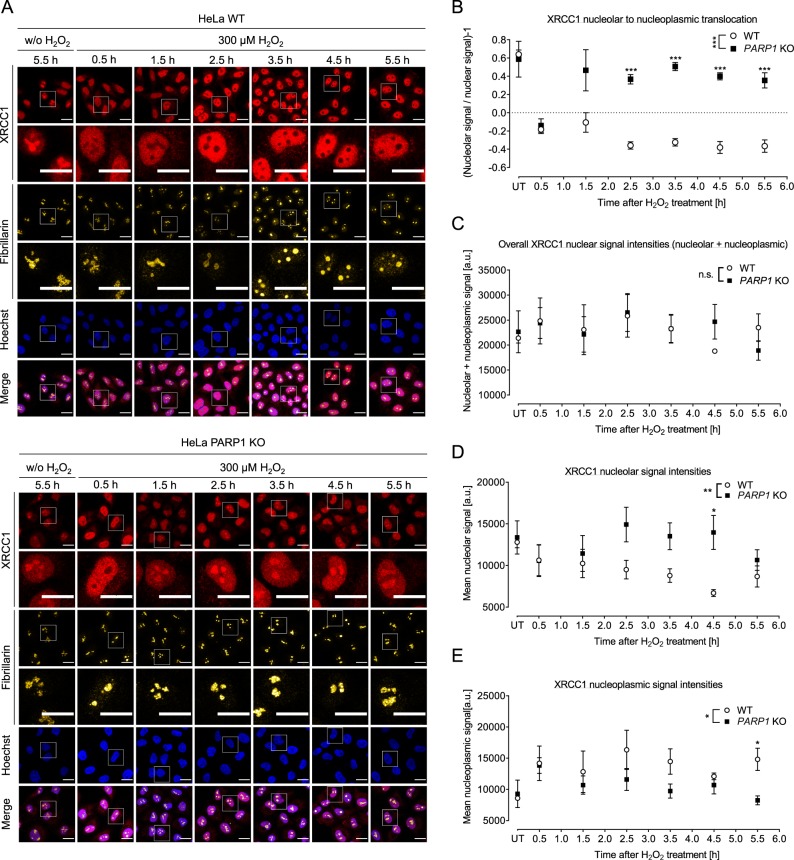
H_2_O_2_-induced XRCC1 translocation from nucleoli to the nucleoplasm is PARP1-dependent. HeLa WT and *PARP1* KO cells were left untreated or treated with 300 µM H_2_O_2_ for periods as indicated. Samples were then subjected to immunofluorescence staining for XRCC1 and fibrillarin and subsequently analyzed by confocal microscopy. White frames indicate magnified image sections. (**A**) Representative images are shown. (**B**) Ratios of XRCC1 nucleolar to nucleoplasmic signal intensities as evaluated by automated image analysis of microscopic data using a KNIME workflow as described in the ‘material and methods’ section and in Suppl. Fig. 8. (**C**) Analysis of overall nuclear signal intensities (nucleolar plus nucleoplasmic signals). (**D**) Analysis of nucleolar signal intensities. (**E**) Analysis of nucleoplasmic signal intensities. Data are means ± SEM of three independent experiments. Statistical analysis was performed by 2-way ANOVA testing. Brightness and contrast were adjusted for better visibility. Scale bars represent 20 µm. ‘UT’ indicates ‘untreated’.

In a next step, we analyzed if H_2_O_2_-induced nucleolar-nucleoplasmic translocation of genome maintenance factors other than WRN are also dependent on PARP1. To this end, we chose XRCC1, which acts as a hub and loading platform during BER and for which a nucleolar-nucleoplasmic translocation was reported previously (see above). Furthermore, we developed an automated image analysis platform based on a KNIME workflow to quantify nucleolar and nuclear immunofluorescence signal intensities of confocal microscopy data (Suppl. Fig. 8A). The image-processing workflow calculates the ratios of the mean fluorescence intensities in nucleolar areas as determined by fibrillarin staining and nucleoplasmic areas as determined by Hoechst 33342 staining (for detailed description see ‘material and methods’ section) (Suppl. Fig. 1B–D). This automated image analysis offers several advantages over the categorized image analysis as mentioned above. For example, it is less time consuming and continuous, quantitative data can be obtained. Consistent with previous reports^18,33^, XRCC1 accumulated in nucleoli of WT cells under non-stress conditions and was released from nucleoli already 30 min after H_2_O_2_ treatment (Fig. 4A,B). By 2.5 h post treatment, most nucleoli completely lacked XRCC1 staining. Interestingly, in *PARP1* KO cells, XRCC1 was also released from nucleoli 30 min after H_2_O_2_ treatment, but by 2.5 h relocalized to nucleoli to the same level as in unchallenged cells. To test if the disappearance of the XRCC1 signal in nucleoli is caused by nucleolar-nucleoplasmic translocation or potentially by protein degradation dynamics, we separately analyzed fluorescence signal intensities in nucleoli and nucleoplasm. As it is evident from Fig. 4C, the sum of signal intensities of XRCC1 in the nucleoli plus the nucleoplasm did not vary significantly throughout the period analyzed. Consistent with the analysis of Fig. 4B, HeLa WT cells showed a significant decrease in XRCC1 nucleolar signals upon H_2_O_2_ treatment, whereas XRCC1 signal intensities in the nucleoplasm increased accordingly, indicating a nucleolar-nucleoplasmic shuttling mechanism of XRCC1 (Fig. 4D,E). In contrast, in HeLa *PARP1 KO* cells nucleolar and nucleoplasmic XRCC1 signal intensities stayed nearly constant, except for the initial release of XRCC1 from nucleoli after 30 min of H_2_O_2_ treatment (compare to Fig. 4C). Since WRN translocation upon CPT was independent of PARP1, we also tested if the same holds true for XRCC1 translocation. Suppl. Fig. 9 demonstrates that XRCC1 is released from nucleoli upon CPT treatment after 5 h in a dose-dependent manner. These results demonstrate that the CPT-induced nucleolar release of XRCC1 is not dependent on PARP1 as it was observed for WRN. In summary, these results revealed that XRCC1, like WRN, also shuttles from nucleoli to the nucleoplasm upon H_2_O_2_ treatment and that this shuttling is also dependent on PARP1. However, the XRCC1 translocation seems to occur in a manner mechanistically distinct from WRN, since kinetic studies indicate that PARP1 is neglectable for XRCC1 release from nucleoli, but necessary for its retention in the nucleoplasm after its release.

### PARylation and PARP1 protein interactions are involved in XRCC1 and WRN subnuclear translocations in a protein-specific manner

**Figure 5:**
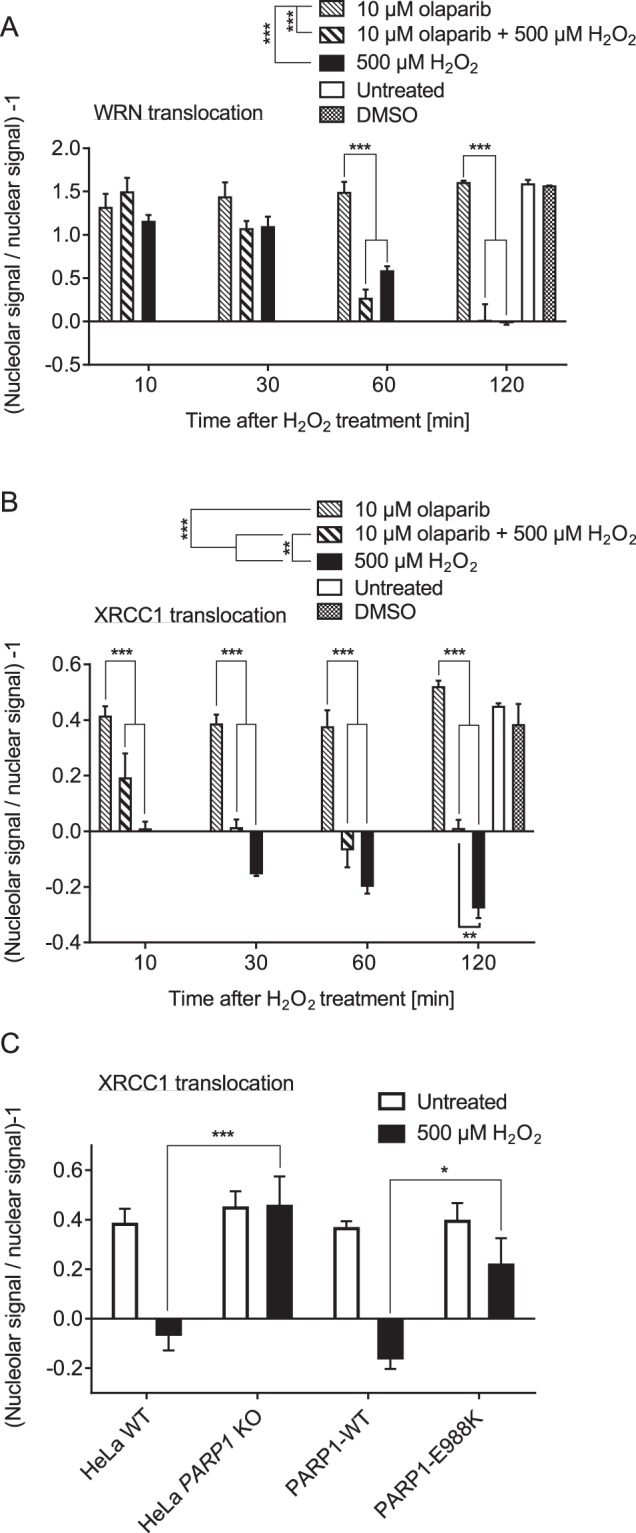
XRCC1, but not WRN translocation from nucleoli, is partially dependent on PARP1 enzymatic activity. (**A**) Quantitative analysis of subnuclear WRN localization using automated image analysis as described in the ‘material and methods’ section. Cells were treated with H_2_O_2_ and the PARP inhibitor olaparib as indicated and subjected to immunofluorescence staining for WRN and fibrillarin. Data represent means ± SEM of three independent experiments. Statistical analysis was performed by 2-way ANOVA testing. (**B**) Quantitative analysis of subnuclear XRCC1 localization. Samples were stained for XRCC1 and fibrillarin, and data presented as described in A. (**C**) HeLa *PARP1* KO cells were reconstituted with PARP1-WT or the mono-ADP-transferase mutant PARP1-E988K, left untreated or treated with 500 µM H_2_O_2_ for 2 h, and subjected to co-immunofluorescence staining for XRCC1 and fibrillarin and subsequent quadruple-color microscopy. Representative images are shown in Suppl. Fig. 10. Shown is the quantitative analysis of subnuclear XRCC1 localization using automated image analysis. Data represent means ± SEM of four independent experiments. Statistical analysis was performed by 2-way ANOVA testing.

To test if WRN and XRCC1 translocations are mediated by PARP1 enzymatic activity we treated WT cells with the clinically relevant PARP1 inhibitor olaparib. As it is evident from Fig. 5A, olaparib did not affect the release of WRN from nucleoli. In contrast, XRCC1 translocation was significantly inhibited by olaparib, leading to a considerable retention of XRCC1 fluorescence signal in nucleoli 2 h post H_2_O_2_ treatment (Fig. 5B). To validate these results, we employed a genetic approach by reconstituting *PARP1* KO cells either with PARP1-WT or a mono-ADP-ribosyl-transferase mutant of PARP1, i.e., PARP1-E988K^19^, and then analyzed XRCC1 translocation upon H_2_O_2_ treatment using quadruple-color immunofluorescence microscopy in combination with automated image analysis (Suppl. Fig. 10). Consistent with the results using pharmacological PARP inhibition, the catalytically compromised PARP1-E988K mutant significantly inhibited the XRCC1 translocation from nucleoli, leading to an intermediate signal intensity of XRCC1 in nucleoli 2 h post H_2_O_2_ treatment (Fig. 5C and Suppl. Fig. 10).

**Figure 6:**
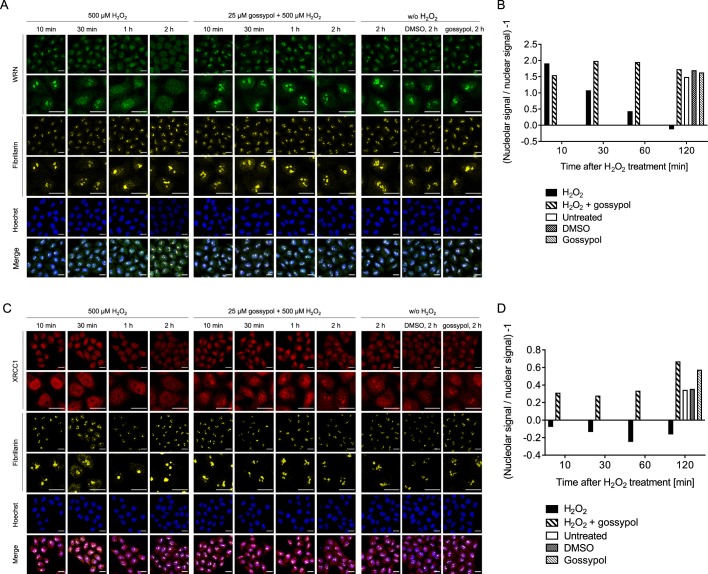
Gossypol inhibits WRN and XRCC1 translocation from nucleoli to the nucleoplasm. (**A**) Subnuclear localization of WRN upon H_2_O_2_-induced DNA damage in the presence of gossypol. Cells were pretreated with 25 µM gossypol for 30 min, then treated with H_2_O_2_ as indicated, and subjected to immunofluorescence staining for WRN and fibrillarin. Representative images are shown. (**B**) Quantitative automated image analysis of microscopic data of A. (**C**) Subnuclear localization of XRCC1 upon H_2_O_2_-induced DNA damage in the presence of 25 µM gossypol. After treatment, samples were subjected to immunofluorescence staining for XRCC1 and fibrillarin, and otherwise processed as in A. Representative images are shown. (**D**) Quantitative automated image analysis of microscopic data of C. Brightness and contrast were adjusted for better visibility. Scale bars represent 20 µm.

Next, we reasoned that interaction of PARP1, which was shown to localize to nucleoli previously^26–28^, may be responsible for the nucleolar-nucleoplasmic translocations of WRN and XRCC1. To test this hypothesis, we used the small molecule gossypol, which was recently shown to inhibit PARP1-protein interactions by targeting its BRCT domain, thereby also inhibiting PARP1’s enzymatic activity^48^. Intriguingly, gossypol treatment completely inhibited the release of both, WRN and XRCC1, from nucleoli upon H_2_O_2_ treatment (Fig. 6). Since gossypol represents a polyphenolic compound with potential antioxidant properties, we performed an H_2_O_2_ scavenging assay. These experiments demonstrated that gossypol in concentration ratios as used in cellular experiments did not exhibit significant H_2_O_2_ scavenging activity (Suppl. Fig. 11).

**Figure 7:**
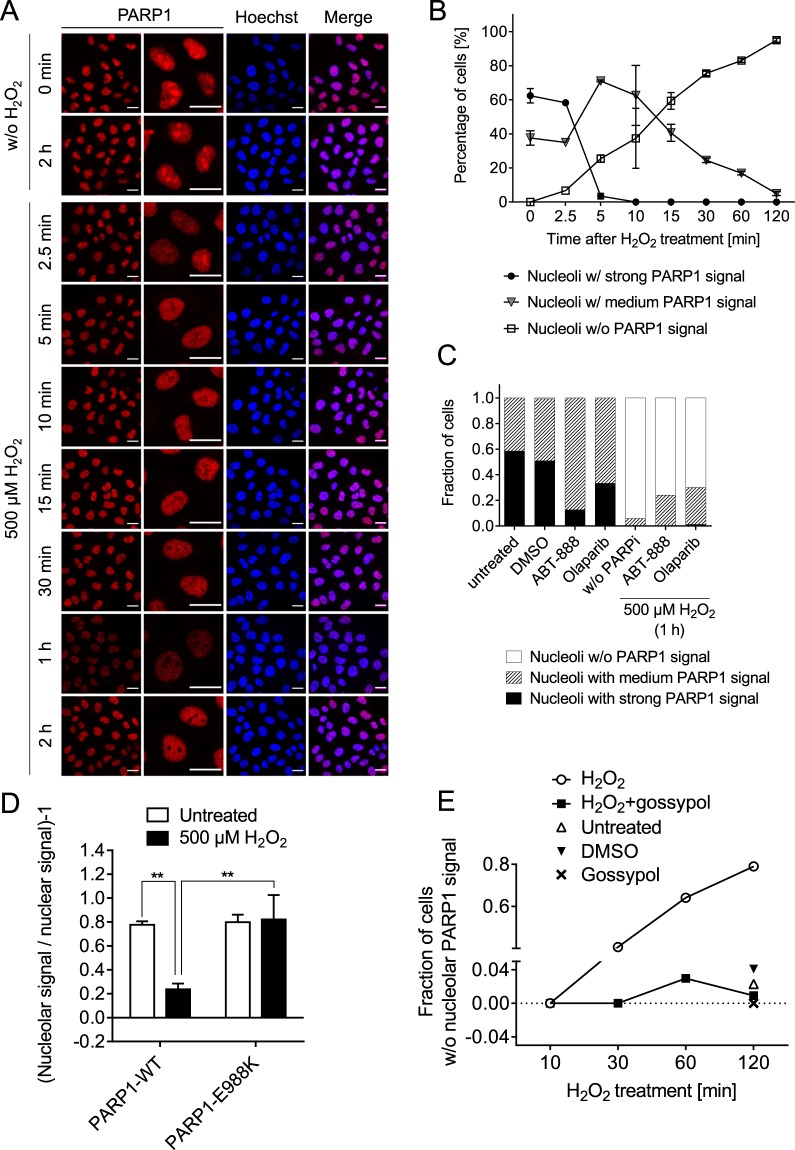
PARP1 translocation from nucleoli to the nucleoplasm upon H_2_O_2_ treatment. (**A**) HeLa cells were treated with H_2_O_2_ for periods as indicated and stained for PARP1. Representative images are shown. Brightness and contrast were adjusted for better visibility. Scale bars represent 20 µm. (**B**) Categorized analysis of nucleolar PARP1 localization as described in the ‘material and methods’ section. (**C**) Effect of PARP inhibitor treatment on PARP1 localization without and with H_2_O_2_ treatment for 1 h. Cells were pretreated with 10 µM ABT888 or olaparib for 30 min. (**D**) HeLa *PARP1* KO cells were reconstituted with PARP1-WT or the mono-ADP-transferase mutant PARP1-E988K, left untreated or treated with 500 µM H_2_O_2_ for 2 h, and subjected to quadruple-color microscopy (c.f. Fig. 5C and Suppl. Fig. 10). Shown is the quantitative analysis of subnuclear PARP1 localization using automated image analysis. Data represent means ± SEM of four independent experiments. Statistical analysis was performed by 2-way ANOVA testing. (**E**) Categorized analysis of subnuclear PARP1 localization upon treatment of cells with 500 µM H_2_O_2_ and pretreatment with 25 µM gossypol for 30 min. Representative microscopic images are shown in Suppl. Fig. 12.

These results prompted us to test if PARP1 itself serves as a shuttle for other proteins to the nucleoplasm upon genotoxic stress. Therefore, we also examined the subnuclear localization behavior of PARP1 upon H_2_O_2_ treatment. As evident from Fig. 7A,B and Suppl. Figs. 10 and 12, PARP1 localized to nucleoli under non-stress conditions and was also quickly released from nucleoli upon H_2_O_2_ treatment. When testing the influence of pharmacological PARP inhibition on PARP1 localization dynamics, it became apparent that on the one hand, PARP inhibitor treatment itself led to a moderate release of PARP1 from nucleoli, potentially by inducing low-level genotoxic stress itself (Fig. 7C). On the other hand, PARP inhibitor treatment resulted in a moderate inhibition of PARP1 translocation from nucleoli upon H_2_O_2_ treatment (Fig. 7C). Consistent with these data are results from the analysis of PARP1 translocation of PARP1-reconstituted cells, which revealed that the mono-transferase mutant PARP1-E988K is retained in nucleoli upon H_2_O_2_ treatment (Fig. 7D and Suppl. Fig. 10). To investigate if PARP1 itself is sufficient for nucleolar-nucleoplasmic shuttling we also tested the effect of gossypol on PARP1 localization. Interestingly, H_2_O_2_-induced PARP1 translocation itself could be completely inhibited by gossypol treatment (Fig. 7E and Suppl. Fig. 12), suggesting that yet unknown factors upstream of PARP1 are at work that trigger the protein release from nucleoli upon genotoxic treatment. It will be interesting to determine this upstream network which leads to a PARP1-dependent release of DNA repair factors such as WRN and XRCC1 in future studies. In summary, these data indicate that WRN and XRCC1 translocations follow distinct mechanisms, since WRN translocation was independent of PARP1 activity, whereas XRCC1 translocation was at least partially dependent on PARP1 activity. Furthermore, these results suggest that additional, yet unidentified upstream factors play a role in these processes, which are involved therein presumably through PARP1-protein interactions.

## Discussion

**Figure 8:**
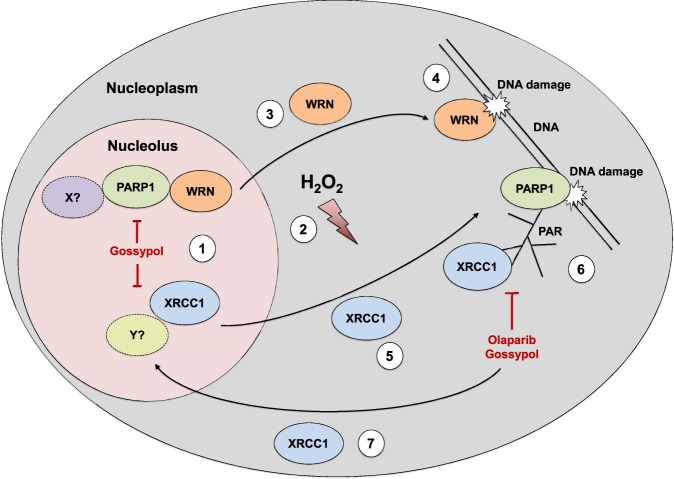
Model summarizing results of this study. (1) Under non-stress conditions considerable fractions of cellular WRN, XRCC1, and PARP1 localize to nucleoli. (2) Upon genotoxic stress, in particular H_2_O_2_ treatment, all three proteins translocate from nucleoli to the nucleoplasm. (3) The translocation of WRN is fully dependent on PARP1 protein, but not on its enzymatic activity. (4) It is conceivable that upon nucleolar release, WRN binds by itself to specific types of DNA lesions or DNA repair intermediates and is thereby retained in the nucleoplasm until its tasks in DNA metabolism are fulfilled. (5) In contrast, a transient XRCC1 translocation can also occur in the absence of PARP1, potentially mediated by an unknown factor ‘Y’. Since XRCC1 by itself exhibits a BRCT domain, which may serve as a binding site to gossypol, it is conceivable that XRCC1 release from nucleoli is also inhibited by this substance. (6) XRCC1 requires DNA damage-bound and PARylated PARP1 as a loading platform, which leads to XRCC1 retention in the nucleoplasm until its tasks in BER are completed. (7) The latter hypothesis is supported by results revealing that in cells without PARP1 activity, XRCC1 relocates quickly to nucleoli.

In this study, we identified PARP1 as a major contributor to genotoxic stress-induced nucleolar-nucleoplasmic shuttling of the two genome maintenance factors, WRN and XRCC1, in HeLa cells. Our findings indicate that PARP1-dependent regulation of nucleolar-nucleoplasmic translocations of WRN and XRCC1 is mediated by distinct mechanisms, some of which are dependent - or independent - of PARP1 enzymatic activity. Our results combined with knowledge from the literature can be summarized by a model as shown in Fig. 8: (*1*) Under non-stress conditions considerable fractions of cellular WRN, XRCC1, and PARP1 localize to nucleoli. For WRN and XRCC1 those nucleolar localizations are largely independent of PARP1, which indicates that PARP1 appears to play no major role in the recruitment of these proteins under non-stress conditions. With regards to WRN, this appears to be regulated by phosphorylation processes^36^. (*2*) Upon genotoxic stress, in particular H_2_O_2_ treatment, WRN translocates from nucleoli to the nucleoplasm. (*3*) The release of WRN from nucleoli is fully dependent on PARP1 protein, but not on its enzymatic activity, indicating a special importance of protein-protein interactions on WRN’s nucleolar localization. This notion is supported by the finding that PARP1 and WRN physically interact via PARP1’s BRCT and WRN’s RQC domain in a PARylation independent manner^43^. Since PARP1 release itself can be inhibited by the PARP1-protein interaction disruptor gossypol, a yet unknown upstream factor ‘X’ may be involved, which controls the release of WRN and PARP1 from nucleoli. [*N.B*. Gossypol inhibits both PARP1-mediated protein interactions as well as PARP1 enzymatic activity via binding to its BRCT domain, whereas olaparib is a strong active-site inhibitor of PARP1 enzymatic activity by binding to its catalytic cleft^48^]. (*4*) It is conceivable that upon nucleolar release, WRN binds by itself to specific types of DNA lesions or DNA repair intermediates^49^ and is thereby retained in the nucleoplasm until its tasks in DNA metabolism are fulfilled. (*5*) In contrast, a fast and transient XRCC1 translocation can also occur in the absence of PARP1, potentially mediated by an unknown factor ‘Y’. The finding that XRCC1 release from nucleoli is also inhibited by gossypol, may be attributed to the fact that XRCC1 by itself exhibits a BRCT domain, which may serve as a binding site for gossypol. (*6*) XRCC1 requires DNA damage-bound and PARylated PARP1 as a loading platform^23,50^, which leads to XRCC1 retention in the nucleoplasm, until its tasks in BER are completed. (7) The latter hypothesis is supported by results revealing that in cells without PARP1 activity, XRCC1 relocates quickly to nucleoli. Taken together, these findings demonstrate that multiple interconnected PARP1-dependent mechanisms are at work controlling protein trafficking between the nucleolus and the nucleoplasm upon genotoxic stress in a very specific manner.

In general, it is assumed that the residence time of nucleolar proteins depends on their affinities to nucleolar anchored hub proteins, such as nucleophosmin or nucleolin, some of which exhibit a nucleolar localization sequence (NoLs). Such proteins may act as carriers or as retention factors for other proteins, which do not necessarily harbor a NoLs by themselves, following the notion that the nucleolar localization in the nucleolus is strongly regulated by protein-protein interactions in a position-dependent and time-dependent manner^18,51^. For example, WRN interacts with nucleolin and this interaction can be dissociated specifically by CPT treatment, but not other agents such as hydroxyurea or mitomycin C^39^. PARP1 is another example of a protein that interacts with such hub proteins, and which by itself does not contain a NoLs. Thus, it was indeed shown that PARP1 interacts with nucleophosmin^28^. This association of PARP1 with nucleophosmin was not dependent on PARP activity and the interaction occurred via the BRCT domain of PARP1. Interestingly, these authors note that PAR formation after H_2_O_2_ treatment is less abundant in the nucleolus, which is consistent with our results (Fig. 1). However, at present it is not clear if this effect is authentic or if it can be potentially attributed to a technical artefact, since PAR formed in the nucleolus is assumed to be very short (around 4–12 subunits)^52^ and the antibody used does not recognize short PAR chains very efficiently. Alvarez-Gonzalez *et al*. demonstrated that such a swift loss of PAR signal upon MNNG treatment could be mediated by caspase-dependent PARP1 cleavage, leading to the retention of the DNA-binding, N-terminal, 29-kDa PARP1 fragment in the nucleolus. In contrast, the potentially highly automodified 85-kDa C-terminal fragment of PARP1 translocates to the nucleoplasm^53^. Furthermore, Guetg *et al*. proposed that nucleolar localization of PARP1 is also dependent on RNA, since treatment of cells with RNase A displaced PARP1 from nucleoli^32^. Of note, the role of PARP1 and PARylation in nucleolar-nucleoplasmic translocations is not unidirectional, since Larsen *et al*. showed that upon DNA damage, NBS1, which acts as a central regulator of DNA damage response, is recruited to nucleoli at least in part in a PARylation-dependent manner^54^. Apart from DNA damage-induced PAR synthesis mainly mediated by PARP1 (and PARP2), a recent study revealed that a PAR-catabolizing enzyme, i.e. TARG1, continuously shuttles between nucleoli and nucleoplasm^55^. Thus, TARG1 localizes to transcriptionally active nucleoli, independently of PAR binding, but in response to DNA damage and activation of PARP1 (and PARP2), TARG1 translocates to the nucleoplasm in a PAR-dependent manner. The results from the present study, taken together with current literature knowledge, support the notion that the role of PARP1 and PARylation in the nucleolus under non-stress as well as under stress conditions is highly complex and that various functionally interrelated and PARP1-associated mechanisms are at work, which strongly depend on the individual protein that undergoes nucleolar-nucleoplasmic shuttling. In this respect, it will be also interesting to study the contribution of other factors, such as potentially PARP2, which may contribute to the residual release of WRN/XRCC1 observed in *PARP1* KO cells.

Another layer of complexity is added by findings showing that liquid-liquid phase separation plays an important role in the internal organization of nucleolar architecture^56^. Interestingly, PAR itself can function as a seed for liquid demixing, in particular in intrinsically disordered proteins, as they are present in nucleoli^57^. It is conceivable that PARylation also regulates the general biophysical state of the nucleolar structure by liquid demixing, thereby contributing to the observed effects. In this regard, it is also worth mentioning that several biophysical parallels exist between assembly of nucleoli and DNA repair foci^58^. Thus, besides protein-specific effects, as shown in the current study for WRN and XRCC1, the intra-cellular compartmentalization, potentially initiated by PAR-dependent phase separation, can contribute to the mechanisms by which PAR is involved in spatio-temporal regulation of both the nucleolar organization and orchestration of DNA repair processes.

A limitation of our study is that, so far, we’ve identified the PARP1 dependence of nucleolar-nucleoplasmic protein shuttling only in HeLa cells. We’ve also tested WT and *PARP1* KO U2OS cells, however, under the conditions tested, we did not observe a PARP1-dependent shuttling effect in this cell type. Since U2OS cells, in contrast to HeLa cells, are p53-positive, it is tempting to speculate that the different behavior may be caused by cell type-specific differences in genotoxic stress response. In future studies it will be important to elucidate for which other cell types and proteins, nucleolar-nucleoplasmic shuttling is dependent on or independent of PARP1 activity and to further decipher the underlying molecular mechanisms of such PARP1-dependent translocations. Furthermore, it will be interesting to address the question how PARP1 and PARylation crosstalk with other post-translational modifications, e.g., acetylation and phosphorylation, which have been shown to be involved in nucleolar-nucleoplasmic trafficking of proteins such as WRN^34,36^. Also, it will be central to dissect DNA repair-dependent and -independent functions of genome maintenance factors on the one hand and ribosomal factors on the other hand, both in the nucleolus and in the nucleoplasm. In conclusion, our results reveal a prominent role of PARP1 in the genotoxic stress-induced nucleolar-nucleoplasmic shuttling of WRN and XRCC1 in a toxicant and protein-specific manner.

## Supplementary information

Supplementary information
