# Supplementary material for: PARP1 regulates DNA damage-induced nucleolar-nucleoplasmic shuttling of WRN and XRCC1 in a toxicant and protein-specific manner
Source: Sci Rep. 2019 Jul 11;9:10075. doi: 10.1038/s41598-019-46358-7 (PMC6624289; doi:10.1038/s41598-019-46358-7)
Supplement: Supplementary file 1 — Supplementary information [file 41598_2019_46358_MOESM1_ESM.pdf]

Supplementary information for:

PARP1 regulates DNA damage-induced nucleolar-nucleoplasmic shuttling of WRN and XRCC1 in a toxicant and protein-specific manner

Sebastian Veith [1, +], Andrea Schink[1, +], Marina Engbrecht[1, +], Matthias Mack[1], Lisa Rank[1], Pascal Rossatti[1], Mariam Hakobyan[1], Denise Goly[1], Tanja Hefele[1], Marco Frensch[1], Arthur Fischbach[1], Alexander Bürkle[1], and Aswin Mangerich[1, *]

[1]Molecular Toxicology Group, Department of Biology, University of Konstanz, Germany,

[+] Shared first authorship

* Correspondence: Aswin Mangerich, Molecular Toxicology Group, Department of Biology, University of Konstanz, D-78457 Konstanz, Germany;
aswin.mangerich@uni-konstanz.de

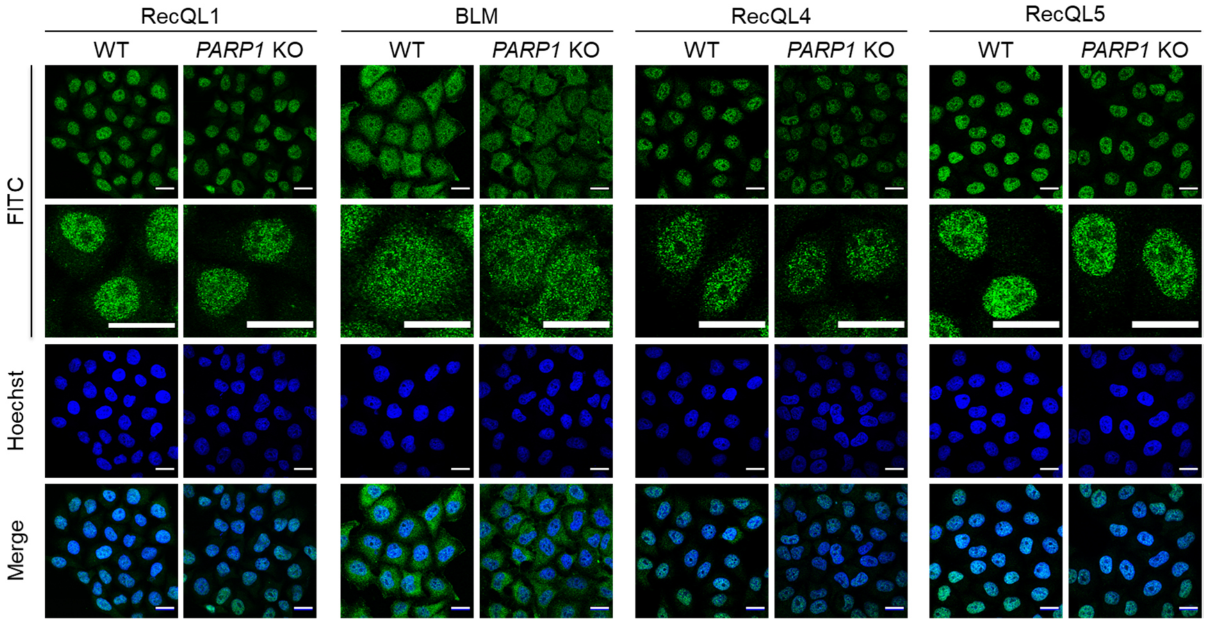

Suppl. Figure 1. Subnuclear localization of RECQ helicases other than WRN. HeLa WT and *PARP1* KO cells were subjected to immunofluorescence staining of RecQL1, BLM, RecQL4, and RecQL5 and subsequent analysis by confocal microscopy. The second row of images represents magnified image sections of the first row. No nucleolar staining of RecQL1, BLM, RecQL4, and RecQL5 could be detected. Representative images are shown. Brightness and contrast were adjusted for better visibility. Scale bars represent 20 µm.

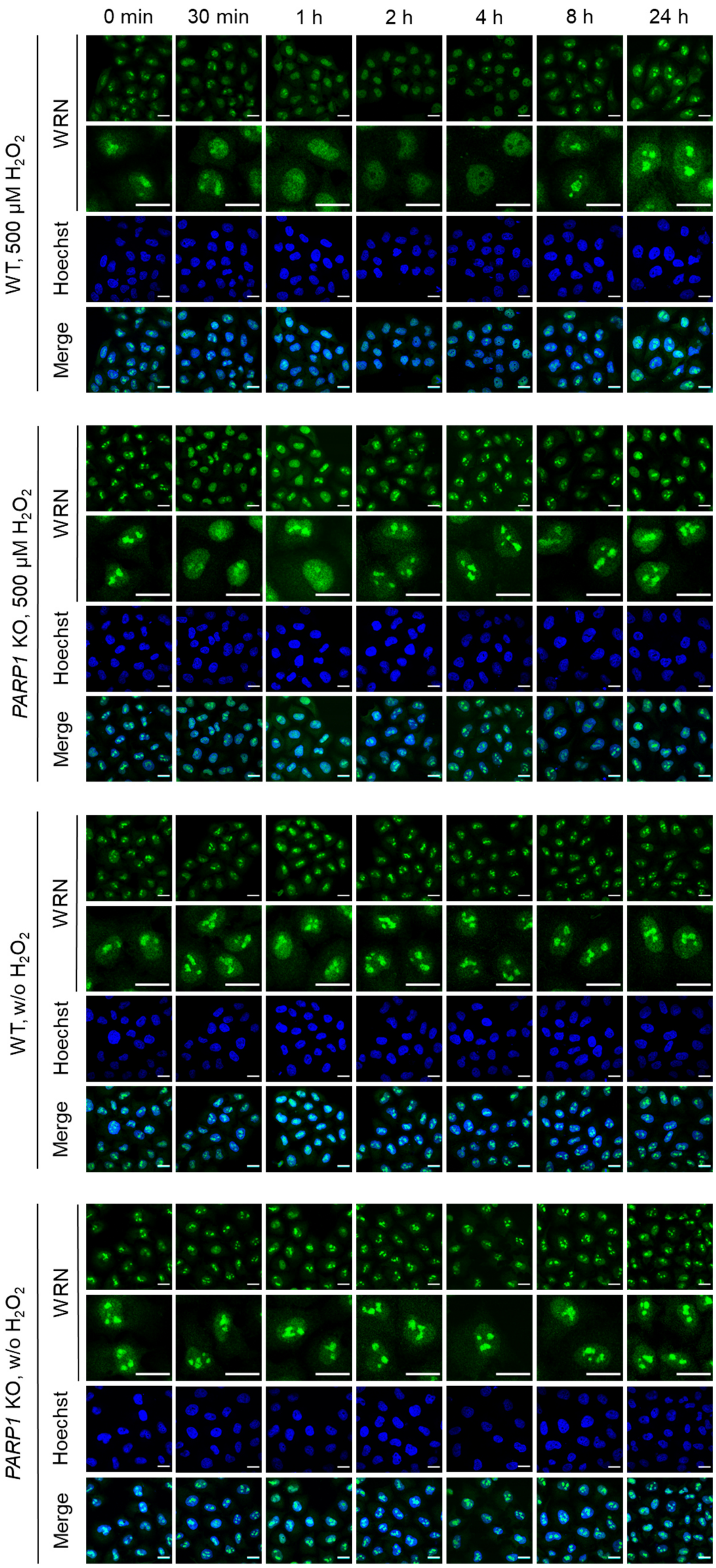

Suppl. Figure 2. H_2O_2-induced PARP1-dependent WRN translocation from nucleoli to the nucleoplasm is fully reversible. Representative microscopic images of data shown in Figure 2. HeLa WT and *PARP1* KO cells were treated as indicated and subjected to immunofluorescence staining for endogenous WRN and analyzed by confocal microscopy. Brightness and contrast were adjusted for better visibility. Scale bars represent 20 µm.

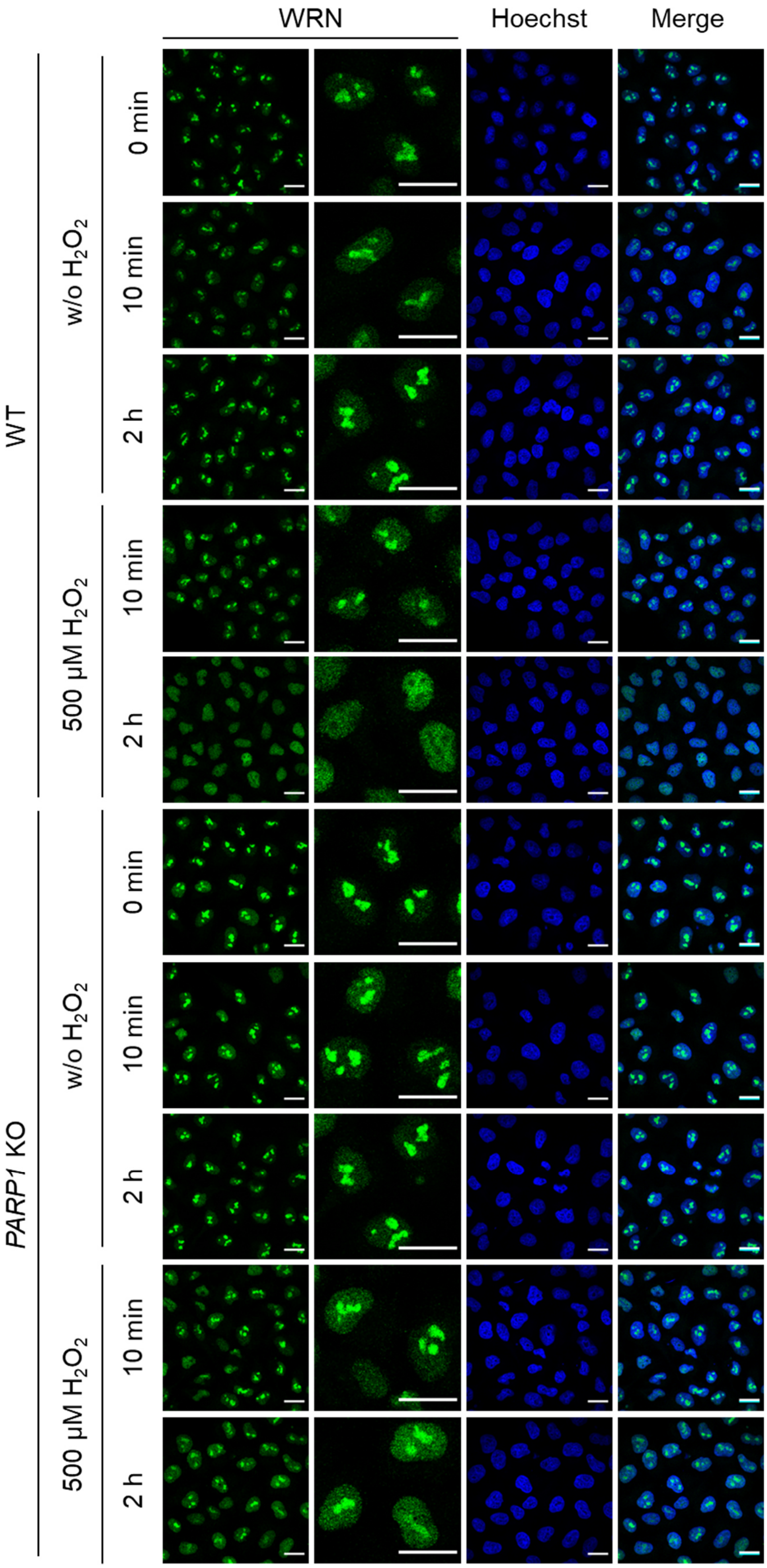

Suppl. Figure 3. Validation of Figure 1 using an alternative WRN-specific antibody. Samples were processed as described in Figure 1. Anti-WRN antibody was a kind gift by Vilhelm Bohr (NIA/NIH). Brightness and contrast were adjusted for better visibility. Scale bars represent 20 µm.

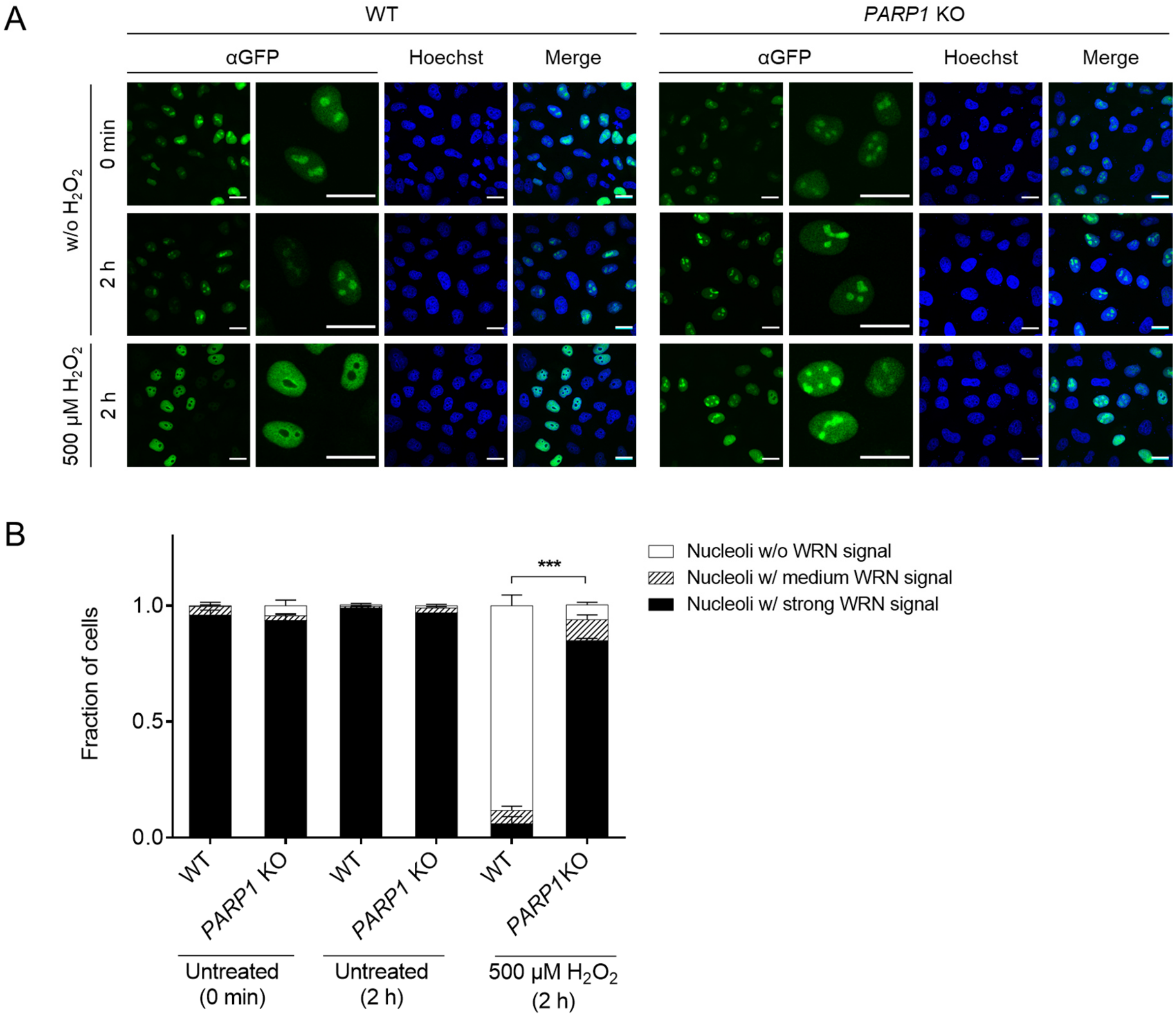

Suppl. Figure 4. H_2O_2-induced nucleolar-nucleoplasmic translocation of ectopically expressed GFP-tagged WRN is PARP1-dependent. One day after transfection of cells with a WRN-eGFP construct, HeLa WT and *PARP1* KO cells were treated with 500 µM H_2O_2 for 2 h as indicated and subjected to immunofluorescence staining for GFP. **A.** Representative images of three independent experiments are shown. **B.** Categorized analysis of WRN localization data as shown in A. Data are means ± SEM from three independent experiments. Statistical evaluation was performed using a Chi square test. Brightness and contrast were adjusted for better visibility. Scale bars represent 20 µm.

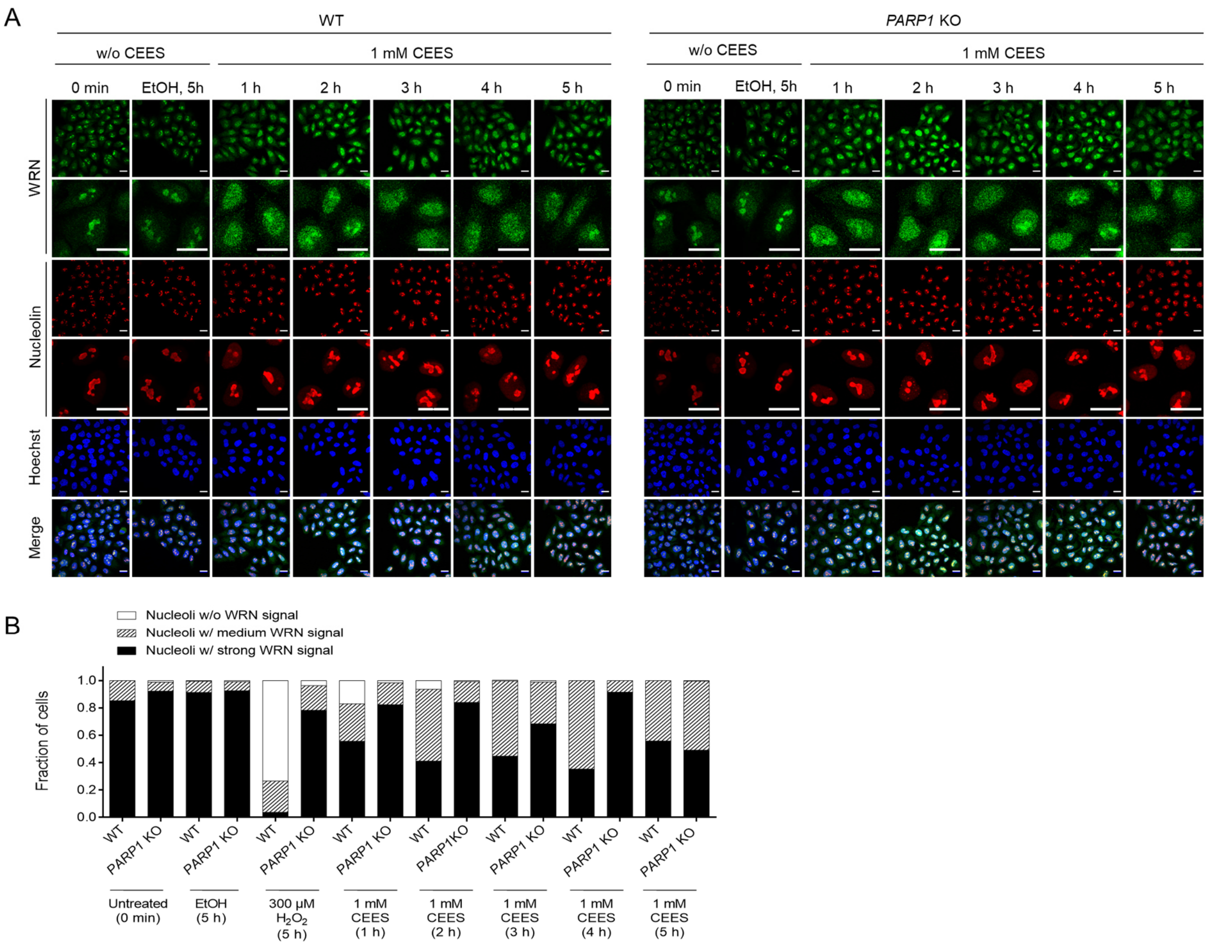

Suppl. Figure 5. CEES-induced WRN translocation from nucleoli to the nucleoplasm is PARP1-dependent. HeLa WT and *PARP1* KO cells were treated as indicated. Samples were then subjected to immunofluorescence staining for WRN and nucleolin and analyzed by confocal microscopy. **A.** Representative microscopic images of the experiment are shown. **B.** Categorized analysis of immunofluorescence data as shown in A. Brightness and contrast were adjusted for better visibility. Scale bars represent 20 µm.

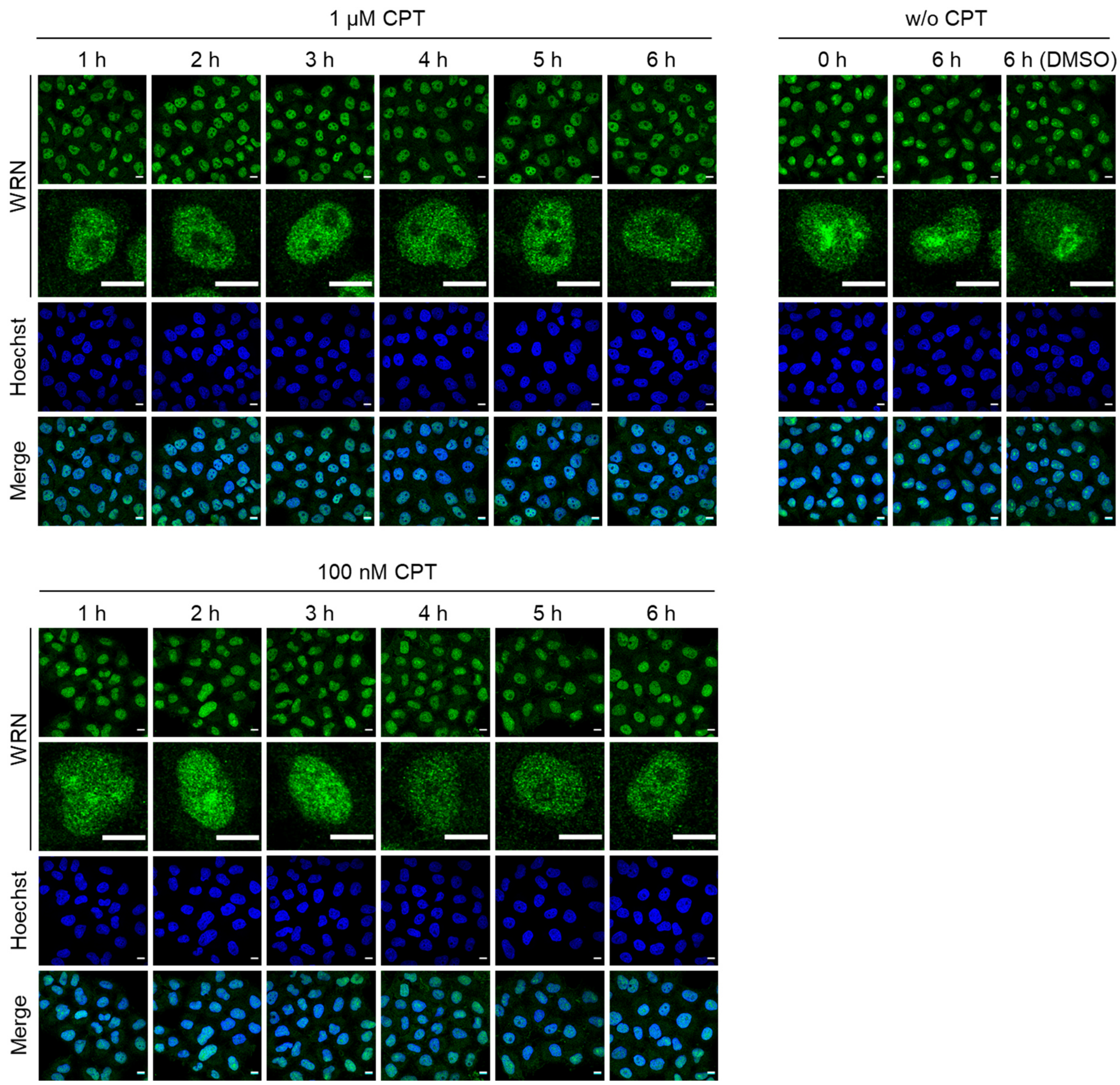

Suppl. Figure 6. CPT-induced WRN translocation from nucleoli to the nucleoplasm. HeLa WT cells were left untreated or treated with camptothecin (CPT) in concentrations and periods as indicated. Samples were then subjected to immunofluorescence staining for WRN and analyzed by confocal microscopy. Second row of images represent magnified section of the first row. DMSO-treated samples served as solvent controls. Brightness and contrast were adjusted for better visibility. Scale bars represent 10 µm.

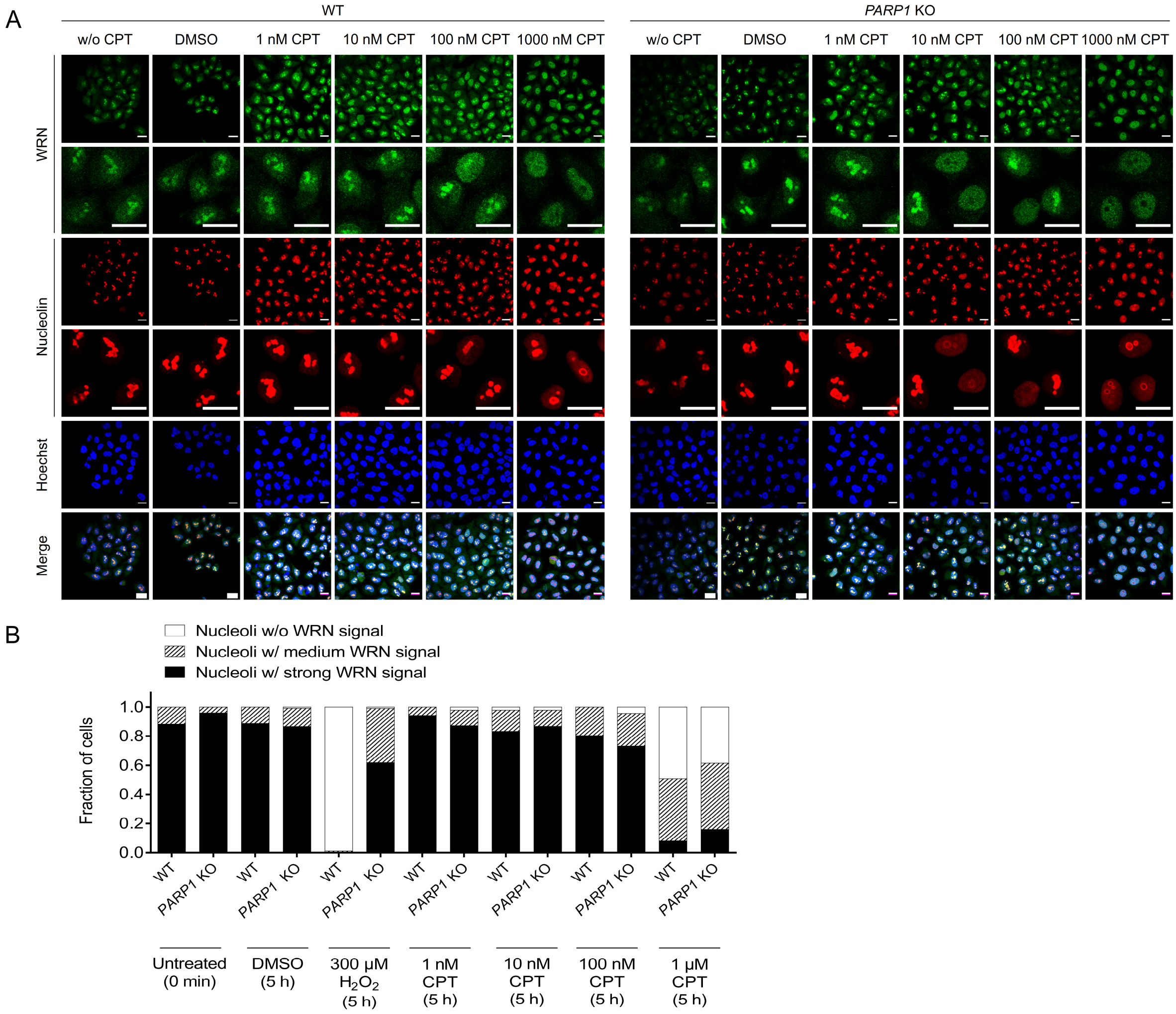

Suppl. Figure 7. CPT-induced translocation of WRN from nucleoli to the nucleoplasm is independent of PARP1. HeLa WT and *PARP1* KO cells were treated with increasing concentrations of camptothecin (CPT) as indicated for 5 h. Samples were then subjected to immunofluorescence staining for WRN and nucleolin and analyzed by confocal microscopy. **A.** Representative microscopic images of the experiment are shown **B.** Categorized analysis of WRN translocation as shown in A. Brightness and contrast were adjusted for better visibility. Scale bars represent 20 µm.

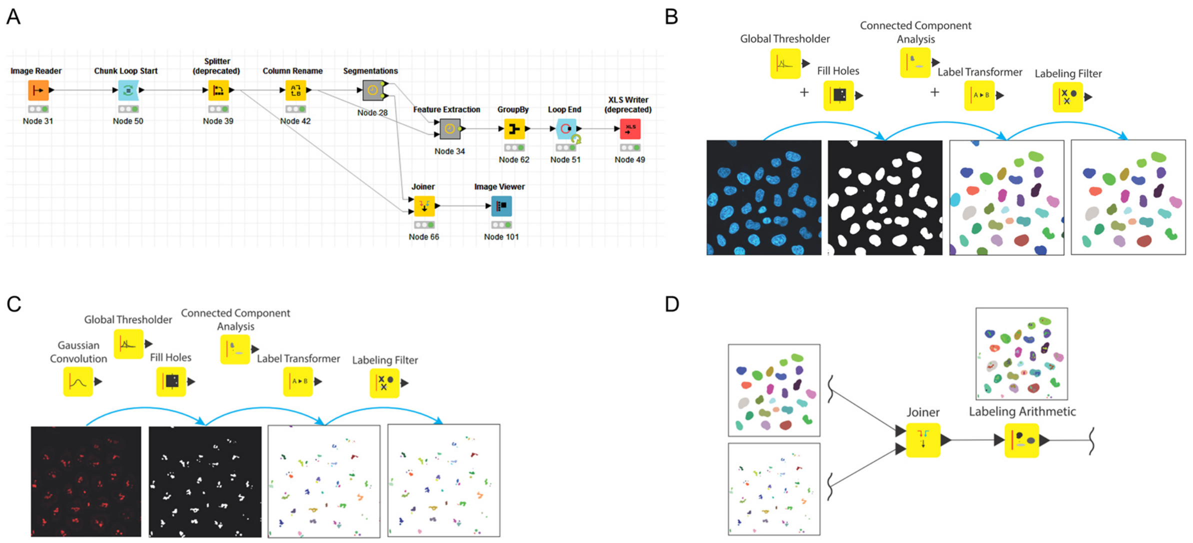

Suppl. Figure 8. KNIME workflow for automated image analysis for nucleolar protein localization. A. Overview of complete KNIME workflow. **B. -D.** Illustration of individual steps of the 'segmentation' meta mode.

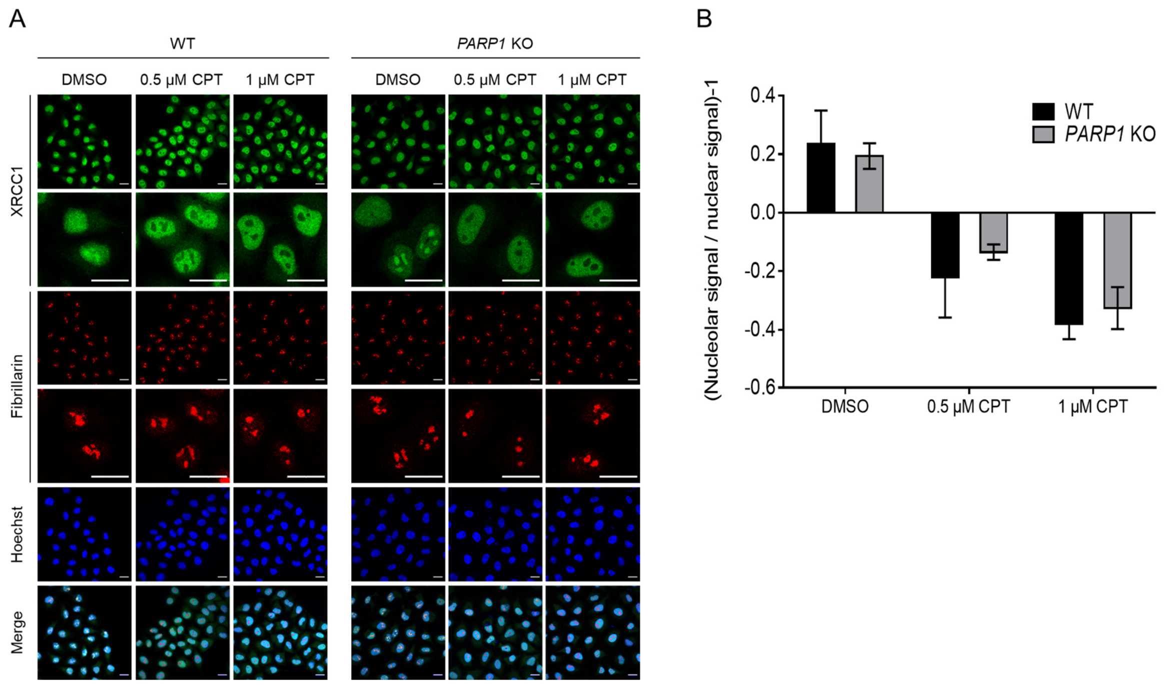

Suppl. Figure 9. CPT-induced translocation of XRCC1 from nucleoli to the nucleoplasm is independent of PARP1. HeLa WT and *PARP1* KO cells were treated with increasing concentrations of camptothecin (CPT) as indicated for 5 h. Samples were then subjected to immunofluorescence staining for XRCC1 and fibrillarin and analyzed by confocal microscopy. **A.** Representative microscopic images of the experiment are shown **B.** Quantitative automated image analysis of microscopic data of A. Brightness and contrast were adjusted for better visibility. Scale bars represent 20 µm.

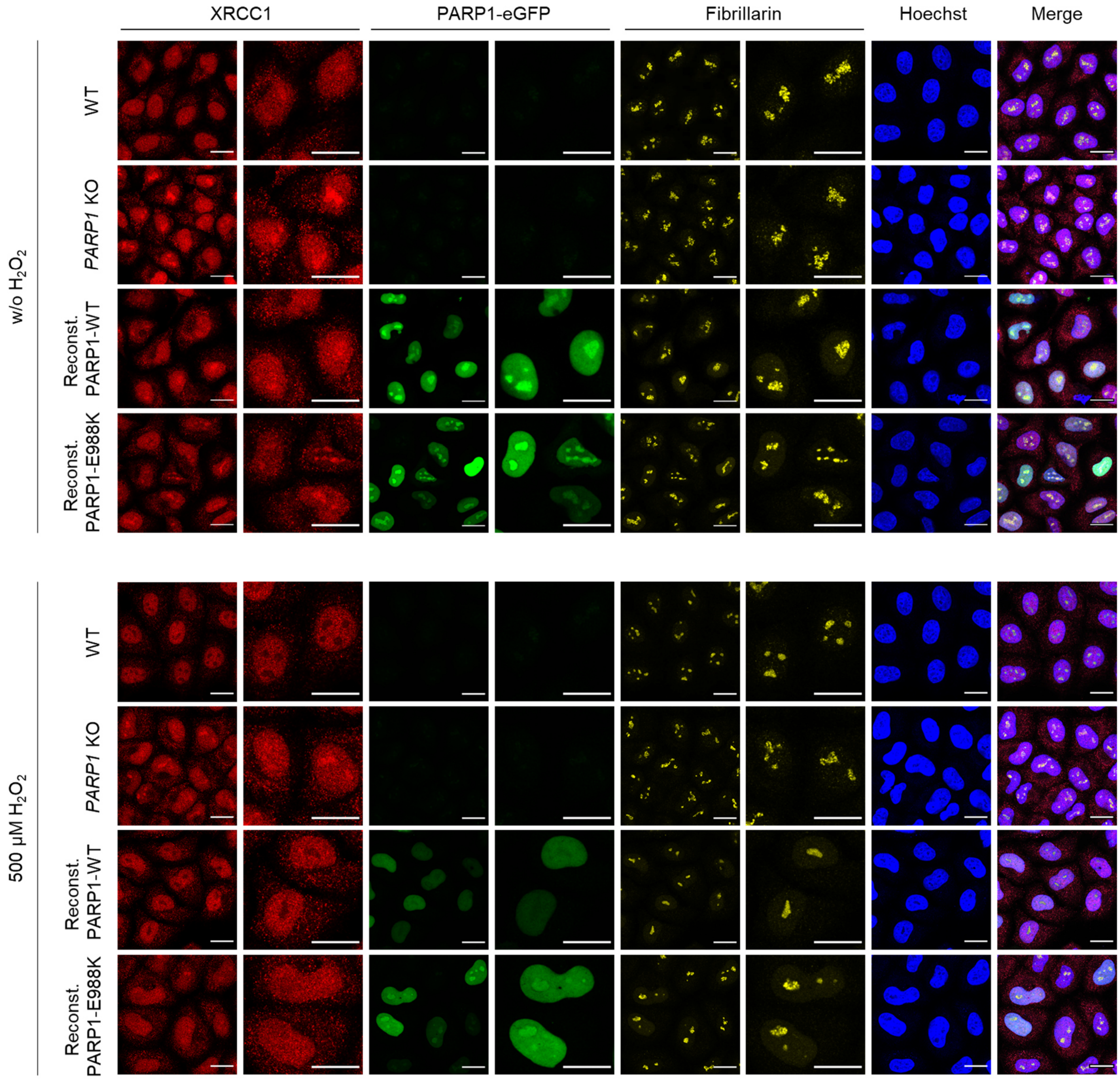

Suppl. Figure 10. XRCC1 translocation is partially dependent on PARP1 enzymatic activity. Representative confocal microscopy images of data presented in **Figure 4 C**. HeLa *PARP1* KO cells were reconstituted with GFP-tagged PARP1-WT or PARP1-E998K as indicated and treated with 500 µM H_2O_2 for 2 h. Then samples were subjected to immunochemical staining for XRCC1 and fibrillarin and analyzed by confocal microscopy. Brightness and contrast were adjusted for better visibility. Scale bars represent 20 µm.

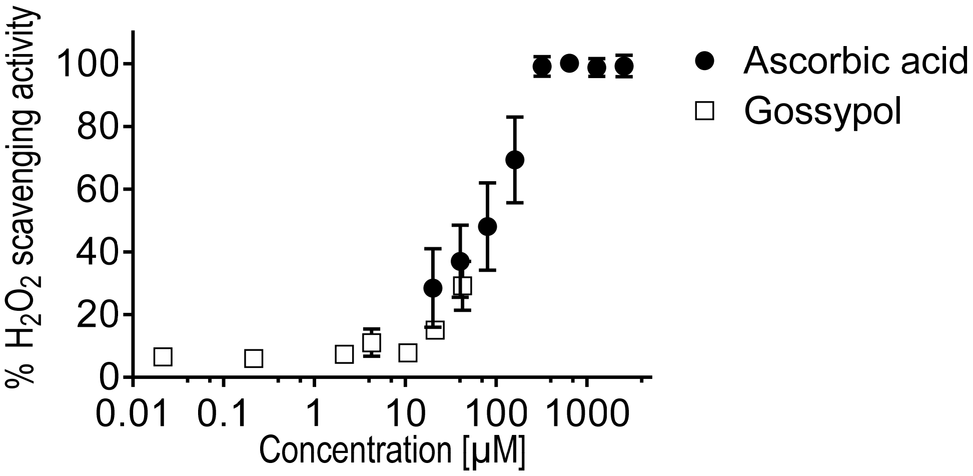

Suppl. Figure 11. H_2O_2 scavenging assay. Gossypol in concentration ratios as used in cellular experiments exhibit no significant H_2O_2 scavenging activity. In this *in vitro* assay, H_2O_2 was used in a final concentration of 94.4 µM. No significant gossypol scavenging activity was evident for gossypol concentrations of up to 21 µM, which refers to a concentration ratio of gossypol to H_2O_2 of '0.22'. In comparison, in cellular experiments, gossypol was used in a concentration of 25 µM together with H_2O_2 concentrations of 300 or 500 µM, which refers to ratios of gossypol to H_2O_2 of 0.08 or 0.05, respectively. Ascorbic acid was used as a positive control. Data represent means ± min/max of two independent experiments.

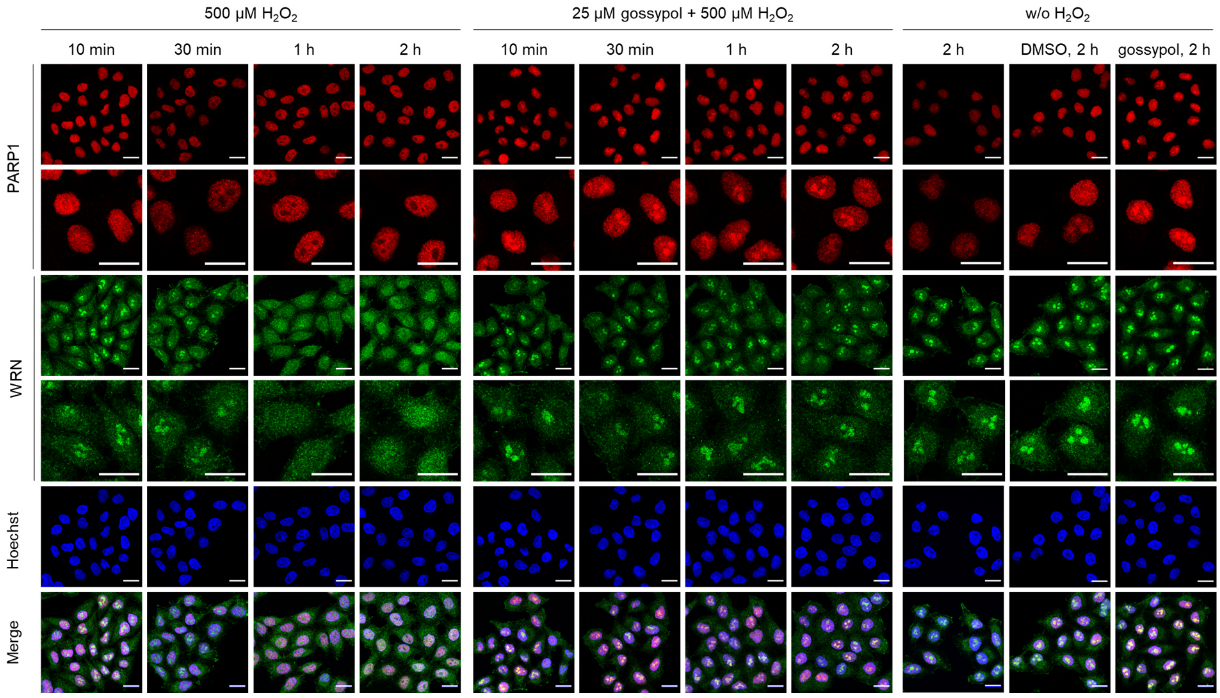

Suppl. Figure 12. PARP1 and WRN translocation from nucleoli is inhibited by gossypol. Representative microscopic images of data presented in **Figure 6 D**. HeLa WT cells were treated with H_2O_2 and gossypol as indicated and subjected to immunochemical staining against PARP1 and WRN and subsequently analyzed by confocal microscopy. DMSO served as solvent control. Brightness and contrast were adjusted for better visibility. Scale bars represent 20 µm.

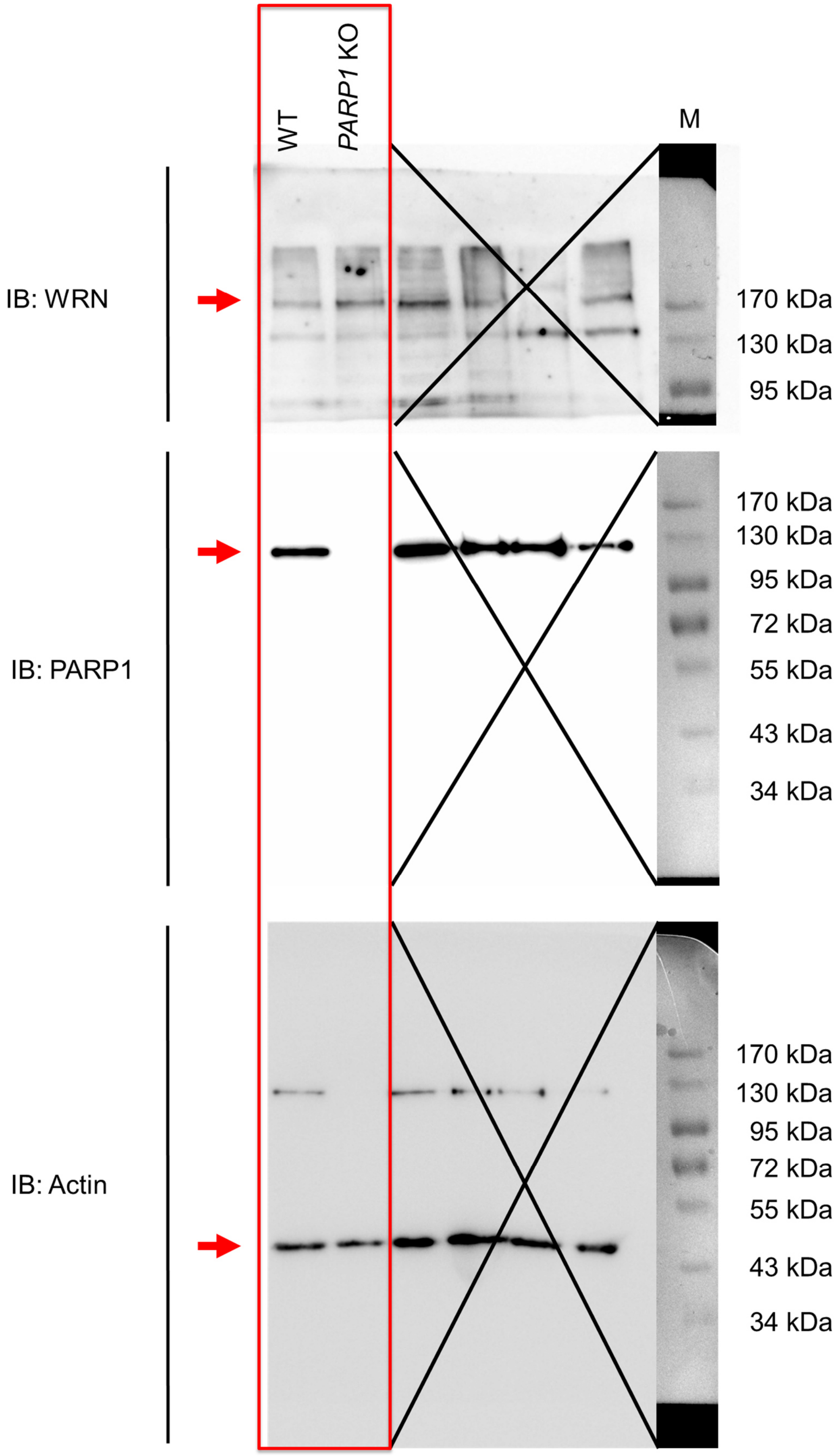

Suppl. Figure 13: Full-length Western blots of Figure 1 A.
